# Identification of the soluble form of tyrosine kinase receptor Axl as a potential biomarker for intracranial aneurysm rupture

**DOI:** 10.1186/s12883-015-0282-8

**Published:** 2015-03-05

**Authors:** Jing Xu, Feiqiang Ma, Wei Yan, Sen Qiao, Shengquan Xu, Yi Li, Jianhong Luo, Jianmin Zhang, Jinghua Jin

**Affiliations:** Department of Neurosurgery, The Second Affiliated Hospital of Zhejiang University School of Medicine, Hangzhou, Zhejiang 310002 China; Department of Neurobiology, Key Laboratory of Medical Neurobiology of the Ministry of Health of China, Zhejiang Province Key Laboratory of Neurobiology, Zhejiang University School of Medicine, 866 Yuhangtang Rd, Hangzhou, Zhejiang 310058 China; Department of Joint Surgery, Shandong Provincial Hospital Affiliated with Shandong University, Jinan, Shandong 250021 China

**Keywords:** Intracranial aneurysm, Rupture, Cerebrospinal fluid, Proteomics, Biomarker, Glycoproteins

## Abstract

**Background:**

Subarachnoid hemorrhage caused by a ruptured intracranial aneurysm (RIA) is a devastating condition with significant morbidity and mortality. Despite the fact that RIAs can be prevented by microsurgical clipping or endovascular coiling, there are no reliable means of effectively predicting IA patients at risk for rupture. The purpose of our study was to discover differentially-expressed glycoproteins in IAs with or without rupture as potential biomarkers to predict rupture.

**Methods:**

Forty age/gender-matched patients with RIA, unruptured IA (UIA), healthy controls (HCs) and disease controls (DCs) (discovery cohort, n = 10 per group) were recruited and a multiplex quantitative proteomic method, iTRAQ (isobaric Tagging for Relative and Absolute protein Quantification), was used to quantify relative changes in the lectin-purified glycoproteins in CSF from RIAs and UIAs compared to HCs and DCs. Then we verified the proteomic results in an independent set of samples (validation cohort, n = 20 per group) by enzyme-linked immunosorbent assay. Finally, we evaluated the specificity and sensitivity of the candidate marker with receiver operating characteristic (ROC) curve methods.

**Results:**

The proteomic findings identified 294 proteins, 40 of which displayed quantitative changes unique to RIA, 13 to UIA, and 20 to IA. One of these proteins, receptor tyrosine kinase Axl, was significantly increased in RIA, as confirmed in CSF from the discovery cohort as well as in CSF and plasma from the validation cohort (*p* <0.05). Spearman’s correlation analysis revealed that the CSF and plasma Axl levels were strongly correlated (r = 0.93, *p* <0.0001). The ROC curve indicated an optimal CSF Axl threshold of 0.12 nM for discriminating RIA from UIA with corresponding sensitivity/specificity of 73.33%/90% and an area under the curve (AUC) of 0.89 (95% CI: 0.80-0.97, *p* < 0.0001). The optimal threshold for plasma Axl was 1.7 nM with corresponding sensitivity/specificity of 50%/80% and an AUC of 0.71 (95% CI: 0.54-0.87, *p* = 0.027).

**Conclusions:**

Both CSF and plasma Axl levels are significantly elevated in RIA patients. Axl might serve as a promising biomarker to predict the rupture of IA.

**Electronic supplementary material:**

The online version of this article (doi:10.1186/s12883-015-0282-8) contains supplementary material, which is available to authorized users.

## Background

Intracranial aneurysms (IAs) are a common type of cerebrovascular disorder with a high prevalence of 1-5% in the general population [1]. Although only 1-2% of IAs rupture annually, such a rupture can cause subarachnoid hemorrhage (SAH), a devastating clinical consequence with high mortality (25-40%) and morbidity (~50%) constituting a major public health problem [2,3]. In contrast, IAs that are repaired before they rupture by microsurgical clipping or by endovascular coiling have a reported mortality as low as 0.6% and morbidity < 5% [4,5]. Therefore, it would be advantageous to discover reliable predictive markers for screening individuals at risk for the development or rupture of an IA.

Recently, much work has focused on identifying susceptibility genes using genome-wide DNA linkage analyses or examining the single-nucleotide polymorphisms of individual genes that might be related to the development of IAs and their subsequent rupture [6-8]. Unfortunately, these studies have frequently yielded conflicting results and associations have not been replicated in other populations despite decades of research. These discrepancies may be due to genetic heterogeneity and a lack of knowledge of the molecular pathogenesis of IA formation, progression, and rupture. The molecular alterations within an aneurysm i.e. the dynamic molecular environment of the vasculature controlled by gene expression, may prove to be more predictive in distinguishing individuals at high risk of IA development or rupture. A series of human and animal studies of IA tissues using gene expression microarrays have highlighted the molecular changes resulting in endothelial dysfunction, the inflammatory response and degeneration of the vascular wall [9]. Although discovering the molecular markers in IA tissue has shed light on pathogenesis, it is neither practical nor feasible to use these molecules as biomarkers for screening individuals at risk for the development or IA rupture unless they can be confirmed in body fluids such as plasma and cerebrospinal fluid (CSF), which are easily accessible and ideal sources for biomarker discovery.

CSF is an important target for proteomic discovery of the disease-specific biomarkers of many brain disorders such as neurodegenerative diseases and traumatic brain injury [10,11]. Several markers have been identified in CSF that could predict susceptibility to aneurysmal SAH and post-SAH cerebral vasospasm [12-14]. However, the comprehensive characterization of CSF is still challenging because of its complexity and the high dynamic range of protein concentrations. To circumvent this difficulty, we focused on one subproteome of CSF: glycosylated proteins. Protein glycosylation is an abundant and biologically important post-translational modification. It is most commonly associated with membrane and secreted proteins. Indeed, many clinical markers and therapeutic targets such as Her2/neu (breast cancer) and prostate-specific antigen (prostate cancer) are glycoproteins [15,16]. In this study, we used a complementary proteomic approach which integrated the lectin-affinity column for glycoprotein enrichment and a multiplex quantitative proteomic method called iTRAQ (isobaric Tagging for Relative and Absolute protein Quantification), to simultaneously quantify relative changes in the glycoproteins of CSF obtained from patients with ruptured IAs (RIAs) and unruptured IAs (UIAs) compared to healthy controls (HCs) and disease controls (DCs). First, we identified a number of proteins that displayed quantitative changes unique to RIA, UIA or IA (discovery cohort, n = 40). Then we confirmed the proteomic results in both CSF and plasma from a larger and independent cohort (validation cohort, n = 80). Finally, we tested the specificity and sensitivity of the candidate marker in CSF and plasma to evaluate whether it could be directly applied as a biomarker of IA rupture.

## Methods

### Characterization of participants

This study was approved by the Medical Ethics Committee of the Second Affiliated Hospital of Zhejiang University School of Medicine (Hangzhou, China), and written informed consent was given by all participants prior to the study. As a discovery step, we selected 40 age/gender-matched RIA, UIA, HC, and DC samples of CSF (n = 10 for each group) from the patients consecutively admitted to the Department of Neurosurgery of the Second Affiliated Hospital from January 2008 to October 2010. We verified the proteomic results of the discovery set using an independent set of 80 age/gender-matched RIA, UIA, HC, and DC samples (n = 20 for each group) from the patients consecutively admitted from January 2008 to October 2012. The demographics are listed in Table 1. The detailed clinical characteristics of all participants are listed in Additional file 1 of the supplementary materials. All study participants were Chinese of the Han ethnic group.

**Table 1:** **Demographics of patients**

**Discovery cohort**					
**Group**	**N**	**Gender (M:F)**	***p*** **value**	**Age (mean ± SD)**	***p*** **value**
RIA	10	8:2		52.4 ± 9.0	
UIA	10	8:2		55.2 ± 3.9	
HC	10	8:2		52.8 ± 6.9	
DC	10	8:2	1^a^	54.6 ± 4.2	0.50^b^
**Validation cohort**					
**Group**	**N**	**M:F**	***p*** ** value**	**Age**	***p*** ** value**
RIA	20	7:13		51.7 ± 8.4	
UIA	20	7:13		51.2 ± 10.2	
HC	20	7:13		50.8 ± 7.6	
DC	20	7:13	1 ^a^	49.5 ± 7.7	0.85^C^

^a^Gender difference between d groups analyzed by the χ^2^ test. ^b^Age difference between groups in the discovery cohort analyzed by the nonparametric Kruskal-Wallis test (the variances were unequal by Bartlett’s test, *p* <0.05). ^c^Age difference between groups in the validation cohort analyzed by the Newman-Keuls multiple comparison test (the variances were equal by Bartlett’s test, *p* >0.05).

Both the discovery and validation samples followed the same inclusion and exclusion criteria. The HCs were selected among patients admitted for cranioplasty who had completely recovered from head trauma for at least six months. The HC inclusion criteria were as follows: 1) all the physical and neurological examination and laboratory tests were within the normal range; 2) no family history of IA and SAH in first-degree relatives; 3) confirmation that they did not harbor IA by digital subtraction angiography (DSA), three-dimensional CT angiography (CTA), or magnetic resonance angiography (MRA); 4) no heavy cigarette-smoking (<2 packs/week) and alcohol use (<50 g/week). The DCs were selected among patients with brain tumors or other brain diseases and it was confirmed that they did not harbor IA by DSA, CTA, or MRA. The RIA group was selected among SAH patients with a diagnosis of IA using DSA. The UIA patients were found incidentally through CTA, MR imaging, or MRA performed for reasons other than suspicion of an index aneurysm and did not present any IA-related clinical symptoms or signs. The most common reasons leading to the discovery of an IA included nonspecific headaches or dizziness or regular physical examination. Most of the UIA patients (28 out of 30) were treated by coiling and clipping. Only 2 UIA patients harboring ophthalmic artery aneurysms underwent follow-up CTA examinations at intervals of 6–12 months.

### Collection of CSF and plasma

Collection of CSF by lumbar puncture was performed as in our previous proteomic study [17]. All CSF samples were collected in the morning after overnight fasting. In addition, all CSF for proteomic analysis was taken from the 15th to 25th ml collected to limit variations arising from the rostral-caudal gradient, and 1.0 mL from each participant was used to generate a pooled sample, that is, a total of 10 ml of CSF by pooling 10 participants for each group. Because the protein concentration in CSF is relatively low compared to plasma (CSF/plasma 1/100-200) and the protein profiles in CSF are similar to those in plasma, even a minor contamination of CSF with blood could significantly confound the interpretation of quantitative or qualitative proteomic analysis. Therefore, the CSF samples from patients who needed endovascular or neurosurgical treatment were collected before the operation. The samples from RIA and some DC patients with cerebral hemorrhage were collected one month after endovascular embolization or neurosurgical operation. The routine CSF biochemistry assays of those specimens (cell count, glucose, and total protein) were all in the normal range and there were <10 RBCs per ml.

Blood samples (10 ml) from the validation cohort were taken by venous puncture in conjunction with lumbar puncture, except for RIA and some DC patients with cerebral hemorrhage. The samples from those patients were collected within 2 h of admission to the hospital. Samples were collected in plastic tubes containing EDTA. Plasma was then separated by centrifugation at 5000 g at 4°C for 15 min, and stored at −80°C in aliquots of 0.l ml per tube until analysis.

### Isolation of glycoproteins from CSF using lectin affinity column

Glycoproteins were isolated from aliquots of pooled CSF samples (2 mL) using a Qproteome Total Glycoprotein kit (Qiagen, Valencia, CA), which contains ConA and WGA lectins and can be used for a general enrichment of the total glycoprotein population from CSF. The sample was dried to ~ 200 μL with a SpeedVac (Thermo, Asheville, NC), then centrifuged at 1000 g for 5 min, the supernatant transferred to another tube, and the pellets dissolved in 100 μL of detergent supplied with the kit. The resultant samples were combined with the binding buffer along with protease inhibitor solution (100×) before they were loaded onto the lectin column according to the manufacturer’s instructions. Finally, the glycoproteins were eluted 6 times with 100 μL of elution buffer with protease inhibitor solution. The pooled eluates were precipitated by adding 4 volumes of ice-cold acetone, incubated at −20°C for 2 h, and then centrifuged at 12,000 g in a pre-cool microcentrifuge at 4°C for 10 min. The supernatant was removed and the air-dried pellet was re-suspended in 0.5 M triethylammonium bicarbonate buffer. The protein concentration was determined by BCA assay (Thermo, Rockford, IL).

### iTRAQ labeling and two-dimensional liquid chromatography

One hundred micrograms of protein from each group (RIA, UIA, HC, and DC) was digested with trypsin and then labeled with one of the four-iTRAQ™ reagents following the manufacturer’s instructions (Applied Biosystems, Foster City, CA). Next, 4 samples labeled with iTRAQ reagents were combined (a total of 400 μg protein), and separated into 6 fractions using a strong cation exchange (SCX) PolySulfoethyl A™ column (2.1 × 200 mm, 5 μm, 300 Å, Poly LC, Columbia, MD).

The 6 SCX peptide fractions were dried down in a SpeedVac, dissolved in 0.5% trifluoroacetic acid, and separated by capillary C18 reverse phase chromatography (Dionex, Sunnyvale, CA). The eluted gradient was mixed with matrix solution and spotted onto a stainless-steel matrix-assisted laser desorption/ionization plate to form a 24 × 24 array for a total of 576 spots using the Probot™ system (LC Packings, Sunnyvale, CA).

### Tandem MS analysis and protein identification and quantification

Quantitative MS/MS analysis was carried out using a 4700 Proteomics Analyzer with TOF/TOF™ Optics (Applied Biosystems) in the reflector positive ion mode. The default calibration was performed before each run, and the mass accuracy calibrated to within 10 ppm using calibration standards. The parameters were set as following: 800–4000 m/z mass range with 1,000 shots per spectrum for MS analysis; a maximum of 15 peaks was selected per spot with a minimum signal-to-noise ratio of 50 and a cluster area of 500 for data-dependent MS/MS analysis. Proteins were identified using the Mascot (Matrix Science, Boston, MA) algorithm and searched against the International Protein Index (IPI; Version 3.10) human protein database from the European Bioinformatics Institute. For false discovery rate (FDR) analysis, the data were searched against the decoy database. A global FDR <1% were included for protein identification. Proteins were quantified by averaging the iTRAQ ratios of all peptides identified. Individual quantification of the identified peptides was based on the individual ratios from signature ion-peak areas of the iTRAQ reagent tags of the identified peptides from RIA, UIA, and DC samples compared with the HC signature ion peak areas. Normalization, using a Gaussian distribution with a median of 1 when all peptides were considered between control and experimental groups, was performed after the iTRAQ ratios were calculated.

### Functional annotation of identified proteins

The functions of the identified proteins were determined by searching against UniprotKB according to the IPI number. UniprotKB is a comprehensive, high-quality, protein database that contains a large amount of information on the biological functions of proteins derived from the research literature.

### Criteria for selecting candidate proteins for further validation

Several criteria were used for validation: 1) >50% increase or decrease ( iTRAQ ratio is > 1.5 or <0.67; 2) markers annotated as glycoproteins with known glycosylation sites or probable/potential glycosylation sites in UniprotKB; 3) markers with a potential contribution to IA formation/rupture; and 4) commercial antibodies available for Western blot or ELISA.

### Validation of Soluble Axl (sAxl) in CSF and plasma by ELISA

Soluble (s) Axl in CSF and plasma were measured with an Axl Human ELISA kit (Abcam, Cambridge, MA) according to the manufacturer’s instructions. Before measurement, CSF and plasma samples were diluted 1:100 and 1:1000 respectively with Assay Diluent A supplied with the kit. The absorbance at 450 nm was read with a microplate reader. The concentration of sAXl in CSF and plasma was determined using a standard curve.

### Statistical analysis

Statistical analysis was performed with GraphPad Prism 5 (La Jolla, CA). The equality of variances was analyzed by Bartlett’s test. One–way analysis of variance followed by the Newman-Keuls multiple comparison test was used for differences between groups when the variances were equal. Otherwise, the nonparametric Kruskal-Wallis test followed by Dunn’s multiple comparison test was used. The correlation between CSF and plasma Axl levels was analyzed by Spearman’s rank-based correlation coefficient. The specificity and sensitivity of the candidate marker were evaluated both graphically and statistically with receiver operating characteristic (ROC) curve methods. *P* <0.05 was considered statistically significant.

## Results

### Identification of glycoproteins in human CSF

**Figure 1:**
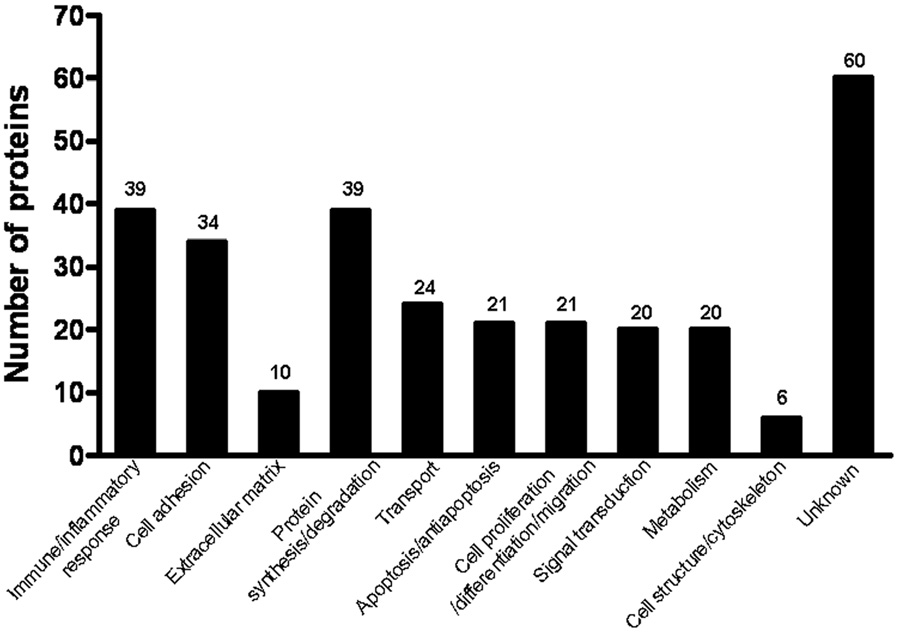
**Functional classification of 294 possible glycoproteins from human CSF.** The proteins were classified into the following categories: immune/inflammatory response, cell adhesion, extracellular matrix (ECM), protein synthesis or protein degradation, transport, apoptosis and anti-apoptosis, cell proliferation/differentiation/migration, signal transduction, metabolism, cell structure/cytoskeleton, and unknown functions. Proteins with multiple functions were assigned to the one that is best known.

By integrating the lectin-affinity purification of glycoproteins with a multiplex iTRAQ quantitative proteomic method, we were able to identify and quantify a total of 294 possible glycoproteins in human CSF with at least two peptides at 95% confidence (Additional file 2). Then we searched all of these proteins against UniprotKB according to the IPI number, and a unique UniprotKB accession number was assigned to each protein (listed in Additional file 3 of the Additional file materials). The functional classification of the identified 294 proteins is shown in Figure 1. This includes immune/inflammatory response, cell adhesion, extracellular matrix (ECM), protein synthesis or protein degradation, transport, apoptosis and anti-apoptosis, cell proliferation/differentiation/migration, signal transduction, metabolism, cell structure/cytoskeleton, and unknown functions. In comparison with UniprotKB, 142 of the 294 proteins were annotated as glycoproteins with known glycosylation sites or probable/potential glycosylation sites. Most of these glycoproteins are secreted or extracellular proteins (Additional file 4).

### Changed glycoproteins in CSF associated with RIA and UIA

In order to determine whether the changes in CSF glycoproteins were specific to IA or common in brain diseases, we had set up a group of DCs including diverse diseases such as brain tumors and cerebrovascular diseases other than aneurysms in addition to the HCs. Therefore, the proteins that were changed in the same direction in both IAs and DCs were excluded. For instance, extracellular superoxide dismutase [Cu-Zn] precursor (IPI00027827), not only displayed an increase in RIAs (iTRAQ ratio 1.60 ± 0.17) but also in DCs (iTRAQ ratio 1.52 ± 0.11) compared to HCs, and would not be considered as a unique change in RIA. However, if a protein such as nectin-like protein 2 (IPI IPI00003813) displayed a significant decrease in RIAs (iTRAQ ratio 0.61 ± 0.13) but an increase in UIAs (iTRAQ ration 1.62 ± 0.23), its change would not only be considered unique to RIA but also to UIA. In addition, a protein would be considered a marker for IA if it displayed a significant change in the same direction in both RIA and UIA. With these criteria, we identified 40 proteins that displayed quantitative changes unique to RIA, 13 to UIA and 20 to IA (Table 2). These changed proteins were found to be involved in processes such as the immune/inflammatory response, cell adhesion, the ECM, protein synthesis and degradation, and apoptosis and anti-apoptosis, which are relevant to the pathogenesis of IA formation and rupture.

**Table 2:** **Protein changes unique to RIA, UIA, or IA**

***Protein name***	***iTRAQ ratios*** ^***a***^			**N** ^**b**^
	**DC**	**RIA**	**UIA**	
**Proteins unique to RIA**				
***Immune/inflammatory response***				
Complement C1s subcomponent precursor	1.03 ± 0.10	**1.28 ± 0.16**	0.96 ± 0.11	4
Pigment epithelium-derived factor precursor	1.29 ± 0.16	**0.76 ± 0.05**	1.22 ± 0.25	4
***Cell Adhesion***				
Alcadein alpha-1	1.22 ± 0.17	**0.81 ± 0.03**	1.32 ± 0.11	2
Calsyntenin-1 precursor	0.78 ± 0.16	**1.20 ± 0.12**	0.82 ± 0.08	3
Contactin 2 precursor	0.93 ± 0.14	**1.20 ± 0.08**	0.95 ± 0.21	5
Nectin-like protein 2	1.09 ± 0.15	**0.61 ± 0.13**	1.62 ± 0.23	3
Neural cell adhesion molecule 1, 140 kDa isoform precursor	0.88 ± 0.12	**0.44 ± 0.14**	0.97 ± 0.21	3
***Extracellular Matrix***				
Secretogranin III precursor	0.83 ± 0.05	**0.63 ± 0.04**	1.00 ± 0.16	3
Splice isoform 1 of fibrinogen gamma chain precursor	0.96 ± 0.15	**1.52 ± 0.19**	1.18 ± 0.12	3
Splice isoform 1 of fibulin-1 precursor	1.03 ± 0.12	**0.64 ± 0.11**	1.09 ± 0.15	3
***Protein synthesis or protein degradation***				
Hypothetical protein FLJ23322	0.64 ± 0.09	**1.43 ± 0.14**	0.46 ± 0.05	2
Hypothetical protein FLJ23757	1.07 ± 0.08	**3.79 ± 0.53**	0.99 ± 0.10	2
PREDICTED: KIAA1509	0.64 ± 0.18	**1.43 ± 0.11**	0.46 ± 0.27	2
Splice isoform 1 of ecto-ADP-ribosyltransferase 3 precursor	1.20 ± 0.16	**3.05 ± 0.31**	1.15 ± 0.23	2
TAR RNA loop binding protein	0.71 ± 0.25	**1.50 ± 0.16**	1.14 ± 0.08	3
***Transport***				
Afamin precursor	1.18 ± 0.08	**1.39 ± 0.05**	1.19 ± 0.12	4
Ceruloplasmin precursor	1.28 ± 0.14	**0.76 ± 0.19**	0.94 ± 0.19	10
Neuronal pentraxin I precursor	1.07 ± 0.10	**0.67 ± 0.04**	0.88 ± 0.03	2
Serotransferrin precursor	0.98 ± 0.47	**0.76 ± 0.50**	0.96 ± 0.35	59
***Apoptosis or anti-apoptosis***				
** AXL receptor tyrosine kinase, isoform 1**	1.12 ± 0.11	**1.68 ± 0.07**	0.87 ± 0.16	2
Splice isoform 1 of amine oxidase flavin containing domain protein 2	0.88 ± 0.13	**0.38 ± 0.14**	1.12 ± 0.08	2
Beta-2-glycoprotein I precursor	0.85 ± 0.14	**1.48 ± 0.20**	1.09 ± 0.09	4
Ephrin A1 isoform b precursor	0.75 ± 0.06	**1.23 ± 0.03**	1.18 ± 0.02	2
Tyrosine phosphatase zeta polypeptide 2 HTPZP2	1.32 ± 0.21	**0.43 ± 0.12**	1.20 ± 0.19	2
***Cell proliferation/differentiation/migration***				
Splice isoform 1 of erythrocyte membrane protein band 4.2	0.93 ± 0.09	**3.86 ± 0.49**	1.53 ± 0.11	2
***Signal transduction***				
XPR1 protein	0.88 ± 0.18	**0.38 ± 0.07**	1.12 ± 0.15	2
***Metabolism***				
Guanylate binding protein 4	1.26 ± 0.13	**0.68 ± 0.10**	1.07 ± 0.11	2
Splice isoform 2 of adenosine kinase	0.88 ± 0.08	**0.38 ± 0.15**	1.12 ± 0.11	2
Splice isoform 2 of phospholipid transfer protein precursor	0.71 ± 0.23	**1.45 ± 0.07**	0.86 ± 0.16	2
***Cell structure/cytoskeleton***				
Nebulin	1.11 ± 0.21	**1.74 ± 0.30**	1.18 ± 0.09	3
PREDICTED: dynein, cytoplasmic, heavy polypeptide 2	0.88 ± 0.09	**0.38 ± 0.12**	1.12 ± 0.16	2
Splice isoform 3 o myosin Va	0.68 ± 0.29	**1.55 ± 0.15**	1.14 ± 0.08	3
Titin	1.08 ± 0.14	**0.64 ± 0.07**	1.07 ± 0.13	2
***Unknown***				
17-beta hydroxysteroid dehydrogenase	0.80 ± 0.21	**1.75 ± 0.23**	1.12 ± 0.16	2
Chromosome 1 open reading frame 27	0.88 ± 0.17	**0.38 ± 0.12**	1.12 ± 0.16	2
JRK protein	1.05 ± 0.05	**1.77 ± 0.17**	0.69 ± 0.06	2
PREDICTED: KIAA1522 protein	0.60 ± 0.16	**1.40 ± 0.20**	0.84 ± 0.12	2
PREDICTED: similar to RIKEN cDNA 1700022C21	0.80 ± 0.21	**1.75 ± 0.16**	1.12 ± 0.10	2
Taste receptor type 2 member 7	0.88 ± 0.12	**0.38 ± 0.06**	1.12 ± 0.04	2
Zinc finger MYND domain containing protein 19	0.97 ± 0.03	**1.31 ± 0.12**	0.86 ± 0.08	2
**Proteins unique to UIA**				
***Immune/inflammatory response***				
Complement C5 precursor	1.34 ± 0.15	1.09 ± 0.07	**0.68 ± 0.05**	2
***Cell Adhesion***				
Splice isoform 1 of neogenin precursor	1.05 ± 0.10	1.10 ± 0.14	**0.71 ± 0.12**	3
***Protein synthesis or protein degradation***				
Hypothetical protein FLJ34458	1.13 ± 0.10	0.94 ± 0.12	**0.63 ± 0.03**	2
SERPIND1 protein	1.01 ± 0.15	1.00 ± 0.08	**1.21 ± 0.06**	4
***Transport***				
Transthyretin precursor	0.95 ± 0.15	0.88 ± 0.07	**0.69 ± 0.11**	5
***Apoptosis or anti-apoptosis***				
Baculoviral IAP repeat-containing protein 4	1.20 ± 0.11	1.00 ± 0.06	**0.47 ± 0.08**	2
Nectin-like protein 2	1.09 ± 0.15	0.61 ± 0.13	**1.62 ± 0.23**	3
Nucleolysin TIAR	0.93 ± 0.07	0.88 ± 0.12	**0.68 ± 0.06**	3
Splice isoform 2 of signal transducer and activator of transcription 1-alpha/beta	1.13 ± 0.06	0.94 ± 0.03	**0.63 ± 0.10**	2
***Cell proliferation/differentiation/migration***				
Condensin subunit 2	0.85 ± 0.10	0.87 ± 0.09	**0.55 ± 0.08**	3
***Signal transduction***				
Grb10 interacting GYF protein 1	1.13 ± 0.05	0.94 ± 0.14	**0.63 ± 0.02**	2
***Metabolism***				
Vitamin D-binding protein precursor	1.14 ± 0.10	1.06 ± 0.18	**1.29 ± 0.09**	6
***Unknown***				
JRK protein	1.05 ± 0.05	1.77 ± 0.17	**0.69 ± 0.06**	2
**Proteins changed both in RIA and UIA**				
***Immune/inflammatory response***				
Complement C3 precursor	0.91 ± 0.40	**1.28 ± 0.42**	**1.25 ± 0.33**	16
Splice isoform 2 of interleukin-17E precursor	1.21 ± 0.09	**0.62 ± 0.13**	**0.67 ± 0.12**	2
***Cell Adhesion***				
Neurocan core protein precursor	0.91 ± 0.15	**0.44 ± 0.10**	**0.54 ± 0.12**	3
Splice isoform 2 of fibrinogen alpha/alpha-E chain precursor	1.14 ± 0.16	**0.56 ± 0.12**	**0.78 ± 0.13**	4
***Extracellular Matrix***				
Extracellular matrix protein 1	1.16 ± 0.12	**0.55 ± 0.15**	**0.69 ± 0.13**	3
Metalloproteinase inhibitor 1 precursor	1.07 ± 0.11	**1.25 ± 0.08**	**1.31 ± 0.12**	4
***Protein synthesis or protein degradation***				
Acheron, isoform 1	0.97 ± 0.03	**1.31 ± 0.08**	**1.64 ± 0.16**	2
***Transport***				
Apolipoprotein D precursor	1.19 ± 0.08	**1.43 ± 0.12**	**1.47 ± 0.11**	3
Serum albumin precursor	1.08 ± 0.17	**1.39 ± 0.11**	**1.59 ± 0.12**	4
***Cell proliferation/differentiation/migration***				
Septin 10 isoform 2	1.08 ± 0.02	**1.28 ± 0.05**	**1.46 ± 0.08**	2
***Signal transduction***				
Adrenomedullin 2 precursor	1.08 ± 0.04	**1.30 ± 0.07**	**1.28 ± 0.04**	2
CD59 glycoprotein precursor	0.90 ± 0.07	**0.49 ± 0.03**	**0.64 ± 0.06**	2
Neuronal pentraxin receptor isoform 1	1.00 ± 0.02	**0.74 ± 0.05**	**0.50 ± 0.11**	3
***Metabolism***				
Lumican precursor	0.99 ± 0.13	**1.57 ± 0.04**	**1.36 ± 0.13**	3
N-acetylgalactosamine-4-O-sulfotransferase	0.88 ± 0.05	**1.21 ± 0.08**	**1.28 ± 0.07**	2
***Cell structure/cytoskeleton***				
Keratin 1	1.49 ± 0.17	**0.66 ± 0.07**	**0.82 ± 0.07**	3
PREDICTED: similar to keratin, type I cytoskeletal 18 (cytokeratin 18) (K18) (CK 18)	1.73 ± 0.28	**0.69 ± 0.06**	**0.76 ± 0.08**	2
***Unknown***				
DJ570F3.6	0.69 ± 0.23	**2.36 ± 0.36**	**2.14 ± 0.24**	2
MUF1 protein	1.09 ± 0.08	**0.72 ± 0.10**	**0.72 ± 0.11**	3
**PREDICTED: hypothetical protein XP_291007**	0.97 ± 0.06	**0.59 ± 0.04**	**0.60 ± 0.04**	2

^a^iTRAQ ratios shown as mean ± S.D. obtained by averaging the iTRAQ ratios of individual peptides. The differences between groups were analyzed by one–way ANOVA followed by Newman-Keuls multiple comparison test. ^b^Numbers of identified peptides used for calculating the iTRAQ ratios. The iTRAQ ratio in bold showed significant differences compared to the other group(s) (*p* < 0.05).

### Validation of sAxl as a potential marker for IA rupture

Given that the current protein database is still incomplete, all the candidate protein markers identified and quantified by proteomics need to be validated before their application in clinical diagnosis is pursued extensively. Therefore we selected several proteins for further validation and Axl was the only one that was not only validated in the discovery CSF samples but also in the validation CSF/plasma samples. Axl, a tyrosine kinase receptor, which displayed a unique increase in the CSF of RIAs, was annotated as an N-linked glycoprotein in the database. A commercial human sAxl ELISA kit was used to validate the iTRAQ assay results.

**Figure 2:**
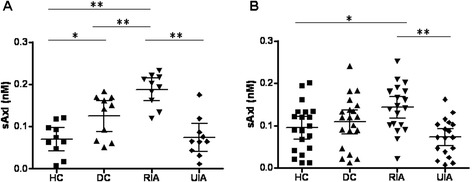
**Confirmation of the changes of CSF sAxl levels in both discovery and validation cohorts using ELISA. (A)** sAxl concentration in individual CSF samples from the discovery cohort (n = 10 per group). **(B)** sAxl concentration in individual CSF samples from the validation cohort (n = 20 per group). *p <0.05 and **p <0.01 (Newman-Keuls test). Error bars represent the 95% confidence interval of the mean.

We first confirmed the changes of sAxl in the pooled CSF samples that were used for proteomic discovery. The ELISA ratio of RIA/HC was 2.35 ± 0.24 while there was no significant difference between UIA *versus*. HC and DC *versus*. HC (Table 3), which was consistent with the iTRAQ results although the relative changes may vary between these two techniques simply because the dynamic range of iTRAQ and ELISA are different. Then we measured the sAxl concentration in the individual CSF samples of the discovery cohort. The results showed that the concentration of sAxl was increased in RIAs as compared to HCs (*p* <0.01), DCs (*p* <0.05), and UIAs (*p* <0.01) (Figure 2A).

**Table 3:** **Comparison of iTRAQ ratio** ***versus*** **ELISA ratio for sAxl in pooled CSF samples**

**Group**	**iTRAQ ratio (mean ± SD)**	**ELISA ratio** ^**a**^ **(mean ± SD)**	**ELISA concentration (nM) (mean ± SD)**
RIA	1.68 ± 0.07	2.35 ± 0.24^*, #, $^	0.204 ± 0.017 ^*, #, $^
UIA	0.87 ± 0.16	0.98 ± 0.47	0.082 ± 0.030
DC	1.12 ± 0.11	1.52 ± 0.48	0.128 ± 0.022
HC	-	-	0.088 ± 0.017

^a^ELISA ratio obtained from 3 independent measurements of sAxl concentration in the RIA, UIA, and DC groups divided by the HC group. The differences between groups were analyzed by one –way ANOVA followed by the Newman-Keuls multiple comparison test. ^*^ *p* <0.01 for RIA *vs* UIA; ^#^ *p* <0.01 for RIA *vs* HC; ^$^ *p* <0.05 for RIA *vs* DC.

**Figure 3:**
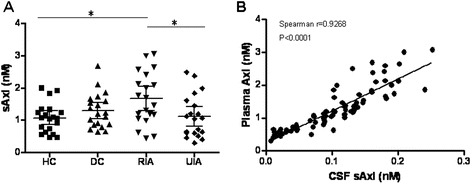
**sAxl concentration in plasma samples from the validation cohort (A) and its correlation with CSF samples (B).** The error bars represent the 95% confidence interval of the mean. The Spearman correlation was significant between plasma and CSF sAxl concentration (r = 0.9268; 95% CI, 0.8865-09532, p <0.0001).

To determine the potential clinical utility of sAxl, we further assessed its levels in a separate larger cohort with paired CSF and plasma samples (n = 20 per group). Compared with CSF, blood analysis has advantages as an approach to population-based disease screening because it is easier and less invasive. In this validation cohort, the mean sAxl of both CSF and plasma was significantly higher in RIAs (CSF, 0.143 ± 0.055 nM; plasma, 1.670 ± 0.802 nM) *versus* HCs (CSF, 0.095 ± 0.057 nM; plasma, 1.073 ± 0.460 nM) and UIAs (CSF, 0.070 ± 0.040 nM; plasma, 1.115 ± 0.659 nM) (Figures 2B and 3A) with levels roughly tenfold higher in plasma, although a wider range of values was found in the validation cohort than in a the discovery cohort, possibly reflecting the heterogeneity of clinical samples. Furthermore, Spearman’s rank-based correlation coefficient analysis showed that CSF and plasma sAxl levels were strongly correlated (r = 0.93, 95% CI 0.87- 0.95, *p* <0.0001; Figure 3B).

**Figure 4:**
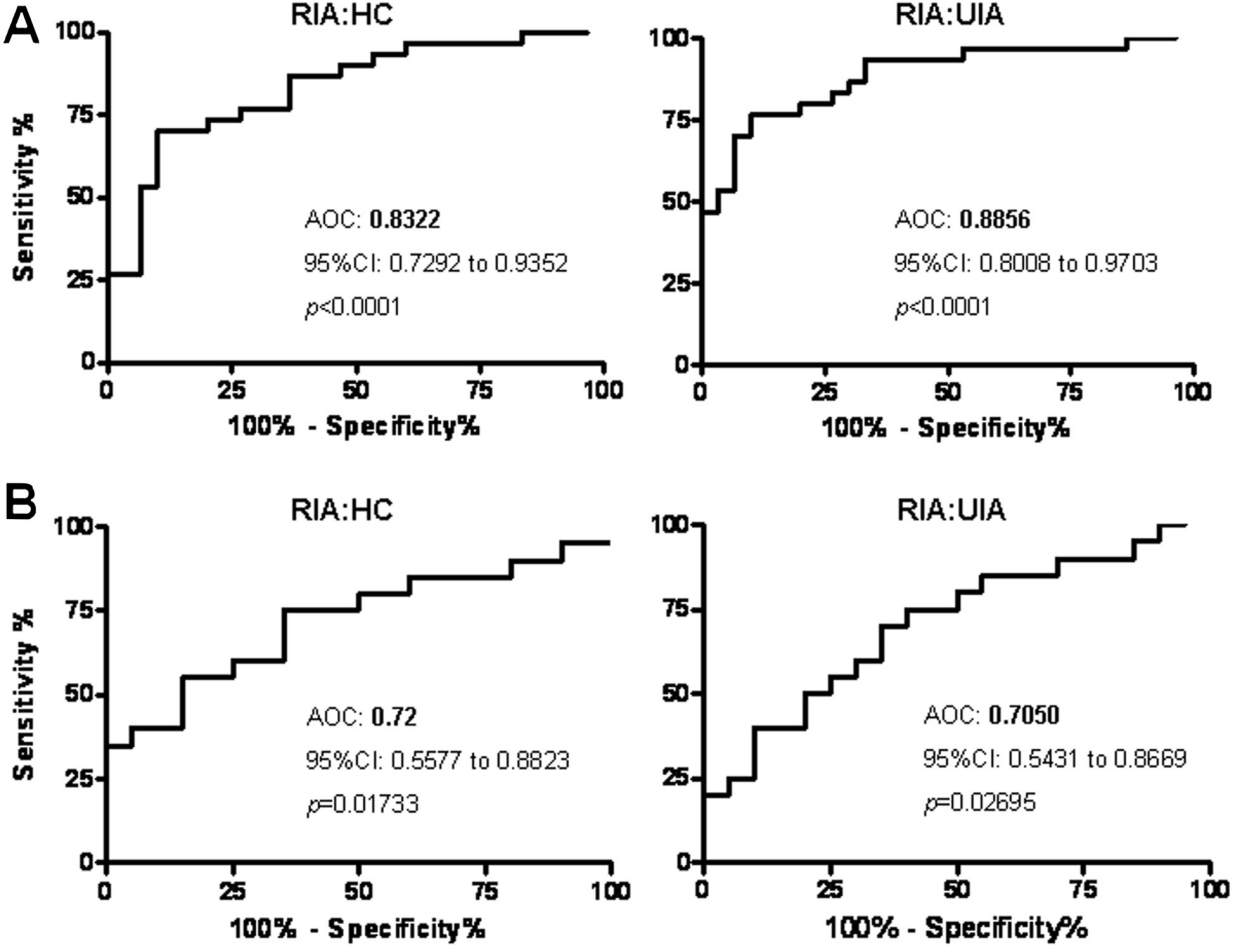
**ROC analysis of RIA** ***versus*** **HC and UIA in CSF (A) and plasma (B).** AUC denotes the area under the ROC curve. *P-*values were analyzed with the Wilcoxon sum-rank test.

Finally, we used the ROC curve to evaluate the sensitivity and specificity of CSF and plasma sAxl in its ability to differentiate RIA from HC, DC, or UIA. The ROC analysis revealed that the CSF sAxl level performed well in discriminating RIA from UIA and HC with areas under the ROC curve (AUCs) of 0.89 (95% CI 0.81-0.97, *p* <0.0001) and 0.83 (95% CI 0.73-0.94, *p* <0.0001) (Figure 4A). The optimal CSF sAxl threshold of 0.12 nM for discriminating RIA from UIA had a corresponding sensitivity/specificity of 73.33%/90% (Table 4). The plasma sAxl level also gave a significant result with AUCs of 0.72 (95% CI 0.56-0.88, *p* = 0.017) and 0.71 (95% CI 0.54-0.57, *p* = 0.027) (Figure 4B). The optimal threshold for plasma sAxl was 1.7 nM with corresponding sensitivity/specificity of 50%/80% (Table 4).

**Table 4:** **Sensitivity and specificity of sAxl in CSF and plasma to discriminate between RIA and UIA**

**sAxl in CSF (n = 30/group)**				
**Cut off (nM)**	**Sensitivity % (95% CI)**	**Specificity % (95% CI)**	**LR+**	**LR-**
0.02	100.00 (88.43 to 100.00)	13.33 (3.76 to 30.72)	1.15	0.00
0.09	90.00 (73.47 to 97.89)	66.67 (47.19 to 82.71)	2.70	0.15
0.10	86.67 (69.28 to 96.24)	70.00 (50.60 to 85.27)	2.89	0.19
0.11	76.67 (57.72 to 90.07)	83.33 (65.28 to 94.36)	4.60	0.28
0.12	73.33 (54.11 to 87.72)	90.00 (54.11 to 87.72)	7.33	0.30
0.14	63.33 (43.86 to 80.07)	93.33 (77.93 to 99.18)	9.50	0.39
**sAxl in plasma (n = 20/group)**				
**Cut off (nM)**	**Sensitivity % (95% CI)**	**Specificity % (95% CI)**	**LR+**	**LR-**
0.4	100 (83.16 to 100.0)	5 (0.1265 to 24.87)	1.05	0.00
0.5	90 (68.30 to 98.77)	15 (3.207 to 37.89)	1.06	0.67
1	80 (56.34 to 94.27)	45 (23.06 to 68.47)	1.45	0.44
1.2	65 (40.78 to 84.61)	65 (40.78 to 84.61)	1.86	0.54
1.7	50 (27.20 to 72.80)	80 (56.34 to 94.27)	2.50	0.63

95% CI, 95% confidence interval; LR+, positive likelihood ratio; LR-, negative likelihood ratio.

## Discussion

Extracellular proteins, including secreted, cell surface and transmembrane proteins are mostly glycosylated and likely to enter the body fluids to serve as potential biomarkers. Lectin-based approaches such as lectin affinity chromatography have been widely used in the discovery of glycoprotein biomarkers in diseases such as hepatocellular carcinoma [18], ovarian tumors [19] and prostate cancer [20]. In the present study, we applied lectin-affinity enrichment of glycoproteins coupled with quantitative iTRAQ-labeling proteomic techniques to discover candidate biomarkers for predicting the risk of development or rupture of IA. Using this quantitative glycoproteomics, we identified CSF proteins either distinctively changed in RIA or UIA or commonly changed in RIA and UIA. The former could be used as candidate biomarkers to predict the risk of IA rupture, while the latter could be used to distinguish individuals at risk of IA formation.

A total of 73 CSF proteins that were differentially expressed in IA compared to controls were identified. These candidate markers fall into all 11 functional categories: 1) immune/inflammatory response, 2) cell adhesion, 3) ECM, 4) protein synthesis or protein degradation, 5) transport, 6) apoptosis and anti-apoptosis, 7) cell proliferation/differentiation/migration, 8) signal transduction, 9) metabolism, 10) cell structure/cytoskeleton, and 11) unknown functions. Among these proteins, some have been implicated in the pathogenesis of IA in previous studies, such as the complement components C1, C3 and C5 [21,22] and the ECM remodeling-related proteins ECM1 and TIMP1 [23]. Furthermore, we also found a number of proteins that have never been reported but are involved in the injury and/or repair of vascular epithelial cells, cell adhesion and migration, and angiogenesis, such as pigment epithelium-derived factor (PEDF) [24], nectin-like protein 2 (NECL2) [25], ephrin A1 [26], neogenin [27], acheron [28], lumican [29] and Axl [30-33].

Of these novel potential biomarkers, receptor protein kinase Axl was distinctively increased in RIA and its differential expression was confirmed in an independent cohort not only in CSF but also in the paired plasma. Axl, which was originally cloned from cancer cells, is broadly expressed in a variety of cells including vascular epithelial cells, smooth muscle cells and fibroblasts [32,33]. Overexpression and an increase of Axl activity have been found in cancer, chronic immune disorders and cardiovascular diseases [30,32,34,35]. Axl-dependent signaling regulates various functions, including survival, growth, aggregation, migration and anti-inflammation, in multiple cell-types. The mechanisms of Axl receptor activation/inactivation are not completely understood. Typically, growth-arrest-specific protein 6 (Gas6), the ligand with the highest affinity for Axl, binds to Axl receptor to form an initial 1:1 Gas6-Axl complex followed by the dimerization of two 1:1 Gas6-Axl complexes, which is a ligand-dependent activation that usually occurs under physiological conditions [32]. During pathophysiological conditions with increased oxidative stress and experimental receptor overexpression, Axl can be activated and autophosphorylated *via* ligand-independent homophilic dimerization [36]. Furthermore, release of a soluble form of Axl (sAxl), the extracellular domain of Axl, is another important feature of Axl receptor biology. A specific proteinase that mediates the cleavage of sAxl has yet to be identified. Axl signaling has mainly been implicated in cancer. However, a rapid increase in publications supports the importance of Axl in other chronic pathological conditions [34,35]. A correlation of plasma concentrations of Gas6 and sAxl with disease activity in systemic lupus erythematosus has recently been reported [31]. Ekam et al. found that plasma Gas6 correlated positively and sAxl correlates negatively with abdominal aortic aneurysms (AAA) size and the Gas6/sAxl ratio may be useful as an AAA biomarker [37]. Nevertheless, more studies are required to determine the role of Axl in the pathogenesis of IA formation, progression, and rupture, and Axl may be a promising biomarker for predicting the risk of IA rupture upon further validation in larger cohorts.

There are some potential limitations in our study. First, we used patients who had completely recovered from head trauma for at least six months as HCs. They may not be considered as “really healthy” controls even if all the results of the physical and neurological examinations and laboratory tests were within the normal range. The longest time period reported in previous studies to analyze inflammatory factors or cytokines in CSF was 22 days and they had returned the normal range by this time [38]. Most of the control patients we recruited had recovered from head trauma for about one year, in order to reduce the effect of disease as much as possible. Second, the CSF samples from the RIA group were collected one month after endovascular embolization or a neurosurgical operation, since contamination of CSF with blood could significantly confound the interpretation of quantitative or qualitative proteomic analysis of CSF. This might not be the ideal time point to collect CSF for RIA. However, considering the inability to collect RIA CSF samples before rupture, it is still reasonable at the discovery step. Moreover, the blood samples of RIA were taken within 2 h after the patients were admitted to hospital. Our results demonstrated that the sAxl level was not only elevated in CSF but also in plasma, which excluded the chance that the increase of sAxl was a result of a restorative process after aneurysm rupture. Third, since most of our UIA patients were treated with coiling or clipping, it is possible that some of them had a risk of rupture. It is interesting that two follow-up UIA patients with ophthalmic artery aneurysms had a relatively low concentration of CSF sAxl (0.076 nM and 0.043 nM). Therefore, next step we will measure the dynamic sAxl levels in CSF and plasma from patients harboring incidental IA and follow-up with CTA every 6 months looking for a correlation between the sAxl levels and the anatomical and/or morphological changes in the aneurysm as signs of impending rupture. So far, we have not recruited enough patients to draw conclusions. Finally, the sensitivity and specificity of plasma sAxl is probably not sufficient to identify RIA patients although the overall expression levels of sAxl from RIA were significantly higher than UIAs and controls. On the one hand, a larger population is needed for further validation. On the other hand, more candidate biomarkers need to be validated.

## Conclusions

In summary, we successfully used lectin-affinity purification and iTRAQ quantitative proteomics approaches to identify candidate biomarkers for predicting the risk of development or rupture of IAs. One candidate biomarker, sAxl, was confirmed to be elevated both in CSF and plasma of RIA patients. The sAxl levels in CSF discriminated RIA from UIA with relatively high sensitivity and specificity, indicating that sAxl might serve as a promising biomarker to predict the rupture of IA.

## Additional files

Additional file 1:
**The detail clinical characteristics of all subjects.**
Additional file 2:
**The peptides identified in human CSF.**
Additional file 3:
**Proteins identified in human CSF.**
Additional file 4:
**Proteins annotated as glycoproteins in UniprotKB.**

## Abbreviations

AAA: Abdominal aortic aneurysms
AUC: The area under ROC curve
CSF: Cerebrospinal fluid
CTA: CT angiography
DC: Disease control
DSA: Digital subtraction angiography
ECM: Extracellular matrix
ELISA: Enzyme-linked immunosorbent assay
Gas6: Growth-arrest-specific protein 6
HC: Healthy control
IA: Intracranial aneurysms
IPI: International Protein Index
iTRAQ: Isobaric Tagging for Relative and Absolute protein Quantification
MRA: Magnetic resonance angiography
MS: Mass spectrometry
RIA: Ruptured intracranial aneurysm
ROC: Receiver operating characteristic
SAH: Subarachnoid hemorrhage
SCX: Strong cation exchange
UIA: Unruptured intracranial aneurysm
