# Supplementary material for: Identification of the soluble form of tyrosine kinase receptor Axl as a potential biomarker for intracranial aneurysm rupture
Source: BMC Neurol. 2015 Mar 5;15:23. doi: 10.1186/s12883-015-0282-8 (PMC4375882; doi:10.1186/s12883-015-0282-8)
Supplement: Additional file 1: — The detail clinical characteristics of all subjects. [file 12883_2015_282_MOESM1_ESM.doc]

**Additional file 1: The detail clinical characteristics of all subjects**

| **ID** | **Age** | **Sex** | **HH** | **FISHER** | **GOS** | **No. Aneurysms** | **Location** | **Size (mm)** |
| --- | --- | --- | --- | --- | --- | --- | --- | --- |
| 1. **10 RIA patients in the discovery cohort** | | | | | | | | |
| **1489459** | 55 | F | 2 | 2 | 5 | 1 | ophthalmic artery | 2.0*1.9 |
| **1597023** | 55 | F | 3 | 3 | 5 | 1 | anterior choroidal artery | 4.5*3.7 |
| **2220083** | 46 | M | 3 | 3 | 5 | 1 | posterior communicating artery | 4.7*4.5 |
| **1814757** | 41 | M | 3 | 3 | 5 | 1 | posterior inferior cerebellar artery | 3.6*3.1 |
| **1346749** | 44 | M | 2 | 3 | 4 | 1 | vertebral artery | 5.6*7.5 |
| **1902485** | 46 | M | 2 | 3 | 5 | 1 | anterior communicating artery | 3.8*3.3 |
| **1417726** | 49 | M | 2 | 3 | 5 | 1 | anterior communicating artery | 4.1*4.3 |
| **1511850** | 55 | M | 1 | 3 | 5 | 1 | anterior communicating artery | 1.9*3.2 |
| **1527347** | 64 | M | 1 | 2 | 5 | 1 | posterior communicating artery | 4.5*3.7 |
| **1745082** | 69 | M | 3 | 4 | 5 | 1 | posterior communicating artery | 3.2*3.3 |
| 1. **20 RIA patients in the valiation cohort** | | | | | | | | |
| **1357563** | 49 | F | 2 | 3 | 5 | 1 | posterior communicating artery | 3.3*3.1 |
| **1550709** | 50 | F | 2 | 3 | 5 | 1 | posterior communicating artery | 7.6*6.3 |
| **1628736** | 51 | F | 1 | 2 | 5 | 1 | posterior communicating artery | 3.5*4.0 |
| **1634389** | 53 | F | 2 | 3 | 5 | 2 | anterior communicating artery, ophthalmic artery | 4.7*4.5  3.0*2.8 |
| **1881083** | 53 | F | 4 | 5 | 3 | 2 | anterior communicating artery  middle cerebral artery | 4.2*5.6  3.1*3.7 |
| **0589107** | 54 | F | 4 | 4 | 3 | 2 | bilateral posterior communicating artery | 4.5*5.1, 3.5*2.7 |
| **1742920** | 48 | F | 3 | 3 | 5 | 2 | anterior communicating artery, cavernous sinus | 4.2*5.4  3.0*4.3 |
| **2757153** | 56 | F | 2 | 3 | 5 | 1 | posterior communicating artery | 4.5*3.4 |
| **2356753** | 62 | F | 2 | 3 | 5 | 2 | bilateral posterior communicating artery | 4.1*4.8, 3.5*3.7 |
| **1900627** | 45 | F | 2 | 2 | 5 | 1 | anterior communicating artery | 3.5*3.2 |
| **2778508** | 55 | F | 2 | 2 | 5 | 1 | posterior communicating artery | 4.3*4.1 |
| **2518933** | 60 | F | 4 | 3 | 4 | 1 | anterior communicating artery | 5.2*4.3 |
| **2302229** | 54 | F | 2 | 3 | 5 | 1 | posterior communicating artery | 4.5*3.6 |
| **2463114** | 59 | M | 2 | 2 | 5 | 1 | anterior communicating artery | 3.1*3.5 |
| **2343531** | 58 | M | 2 | 2 | 5 | 1 | posterior communicating artery | 7.8*6.5 |
| **2254106** | 38 | M | 3 | 4 | 5 | 1 | anterior inferior cerebral artery | 6.3*5.5 |
| **1831133** | 65 | M | 2 | 3 | 5 | 1 | anterior communicating artery | 2.8*3.7 |
| **1584872** | 43 | M | 2 | 3 | 4 | 1 | anterior communicating artery | 4.0*4.5 |
| **2256929** | 51 | M | 2 | 4 | 5 | 1 | anterior communicating artery | 3.7*4.3 |
| **1679664** | 29 | M | 2 | 3 | 5 | 1 | posterior communicating artery | 3.5*3.0 |
| 1. **10 UIA patients in the discovery cohort** | | | | | | | | |
| **1891242** | 56 | F | 0 | 0 | 5 | 1 | ophthalmic artery | 3.1*2.4 |
| **1945308** | 55 | F | 0 | 0 | 5 | 2 | bilateral posterior communicating artery | 5.0*4.2, 3.2*2.7 |
| **1898603** | 47 | M | 0 | 0 | 5 | 1 | anterior communicating artery | 8.2*6.8 |
| **1584121** | 52 | M | 0 | 0 | 5 | 1 | anterior cerebral artery | 3.5*6.3 |
| **2497286** | 53 | M | 0 | 0 | 5 | 1 | basilar artery | 21*23 |
| **1997596** | 56 | M | 0 | 0 | 5 | 1 | ophthalmic artery | 2.5*2.3 |
| **1971267** | 56 | M | 0 | 0 | 4 | 2 | basilar artery  posterior communicating artery | 3.0*2.3  2.2*2.7 |
| **1335408** | 58 | M | 0 | 0 | 5 | 1 | posterior communicating artery | 4.0*5.5 |
| **0611411** | 60 | M | 0 | 0 | 5 | 2 | posterior communicating artery, ophthalmic artery | 3.1*3.4,  4.2*4.6 |
| **1607656** | 60 | M | 0 | 0 | 5 | 1 | vertebral artery | 6.2*8.5 |
| 1. **20 UIA patients in the validation cohort** | | | | | | | | |
| **2241402** | 45 | F | 0 | 0 | 5 | 1 | middle cerebral artery | 3.5*3.7 |
| **2095917** | 44 | F | 0 | 0 | 5 | 2 | vertebral artery,  internal carotid artery | 6.5*7.3  3.2*4.5 |
| **0610352** | 50 | F | 0 | 0 | 5 | 2 | posterior communicating artery, vertebral artery | 5.0*5.8  8.0*11 |
| **2010366** | 51 | F | 0 | 0 | 5 | 1 | ophthalmic artery | 24*21 |
| **0896788** | 54 | F | 0 | 0 | 5 | 1 | posterior communicating artery | 2.6*2.5 |
| **1684651** | 54 | F | 0 | 0 | 5 | 2 | bilateral ophthalmic artery | 4.9*5.2, 3.2*4.1 |
| **2156622** | 60 | F | 0 | 0 | 4 | 1 | ophthalmic artery | 16*23 |
| **0592006** | 63 | F | 0 | 0 | 5 | 1 | posterior communicating artery | 11*8.5 |
| **2145060** | 59 | F | 0 | 0 | 4 | 1 | bilateral posterior communicating artery | 8.9*7.5,5.0*5.5 |
| **2932578** | 43 | F | 0 | 0 | 5 | 1 | posterior inferior cerebellar artery | 3.1*3.5 |
| **2373298** | 46 | F | 1 | 2 | 5 | 3 | choroidal artery  ophthalmic artery  posterior communicating artery | 3.2*3.5  4.5*3.6  3.7*2.8 |
| **2763364** | 58 | F | 0 | 0 | 5 | 1 | ophthalmic artery | 8.5*7.6 |
| **2175423** | 60 | F | 0 | 0 | 5 | 1 | internal carotid artery | 7.9*6.5 |
| **2311733** | 61 | M | 0 | 0 | 5 | 2 | anterior communicating artery  middle cerebral artery | 4.6*5.2  3.5*4.1 |
| **2693352** | 57 | M | 0 | 0 | 5 | 1 | vertebral artery | 6.3*9.1 |
| **2208799** | 37 | M | 0 | 0 | 4 | 2 | bilateral ophthalmic artery | 8.0*6.8, 5.3*4.7 |
| **2641568** | 24 | M | 0 | 0 | 4 | 1 | middle cerebral artery | 5.0*6.8 |
| **2143620** | 43 | M | 0 | 0 | 4 | 1 | middle cerebral artery | 21*17 |
| **1674700** | 56 | M | 0 | 0 | 5 | 1 | middle cerebral artery | 7.4*5.9 |
| **1378221** | 64 | M | 0 | 0 | 5 | 3 | posterior communicating artery  middle cerebral artery  ophthalmic artery | 3.6*4.7  5.1*5.6  9.4*8.7 |
| 1. **10 HC in the discovery cohort** | | | | | | | | |
| **ID** | **Age** | **Sex** | **GOS** | **Operation history** | | **Cerebral hemorrhage** | | **Cerebral infarction** |
| **1355177** | 55 | F | 5 | cranioplasty | | None | | None |
| **1821925** | 53 | F | 5 | cranioplasty | | None | | None |
| **0612187** | 52 | M | 5 | cranioplasty | | None | | None |
| **1656683** | 63 | M | 5 | None | | None | | None |
| **0589337** | 53 | M | 5 | cranioplasty | | None | | None |
| **1935983** | 54 | M | 5 | cranioplasty | | None | | None |
| **1551664** | 60 | M | 5 | None | | None | | None |
| **2071999** | 55 | M | 5 | cranioplasty | | None | | None |
| **0589337** | 43 | M | 5 | cranioplasty | | None | | None |
| **2751666** | 40 | M | 5 | cranioplasty | | None | | None |
| 1. **20 HC in the validation cohort** | | | | | | | | |
| **1625250** | 45 | F | 5 | cranioplasty | | None | | None |
| **1726244** | 48 | F | 5 | cranioplasty | | None | | None |
| **1704862** | 33 | F | 5 | cranioplasty | | None | | None |
| **2476536** | 48 | F | 5 | None | | None | | None |
| **0745031** | 52 | F | 5 | None | | None | | None |
| **2204894** | 54 | F | 5 | cranioplasty | | None | | None |
| **2230835** | 54 | F | 5 | cranioplasty | | None | | None |
| **0976467** | 60 | F | 5 | cranioplasty | | None | | None |
| **2103299** | 61 | F | 5 | cranioplasty | | None | | None |
| **1108068** | 49 | F | 5 | cranioplasty | | None | | None |
| **0867392** | 57 | F | 5 | cranioplasty | | None | | None |
| **1696388** | 62 | F | 5 | cranioplasty | | None | | None |
| **0888267** | 44 | F | 5 | cranioplasty | | None | | None |
| **1863929** | 36 | M | 5 | cranioplasty | | None | | None |
| **0558883** | 56 | M | 5 | cranioplasty | | None | | None |
| **1809989** | 58 | M | 5 | cranioplasty | | None | | None |
| **1588821** | 48 | M | 5 | cranioplasty | | None | | None |
| **0987505** | 49 | M | 5 | cranioplasty | | None | | None |
| **0587762** | 50 | M | 5 | cranioplasty | | None | | None |
| **0612033** | 51 | M | 5 | cranioplasty | | None | | None |
| 1. **10 DC in the discovery cohort** | | | | | | | | |
| **ID** | **Age** | **Sex** | **GOS** | **Disease** | | **Cerebral hemorrhage** | | **Cerebral infarction** |
| **0589100** | 54 | F | 5 | acoustic neuroma | | None | | None |
| **0589581** | 56 | F | 5 | meningioma | | None | | None |
| **0587809** | 58 | M | 5 | pituitary tumor | | None | | None |
| **0581307** | 53 | M | 5 | temporal tumor | | None | | None |
| **0588610** | 55 | M | 5 | frontal tumor | | None | | None |
| **1190128** | 49 | M | 5 | meningioma | | None | | None |
| **2803562** | 52 | M | 5 | arteriovenous malformation | | None | | None |
| **1629929** | 64 | M | 5 | vascular malformation (brainstem) | | None | | None |
| **0578814** | 54 | M | 5 | intracranial tumor | | None | | None |
| **1501053** | 51 | M | 5 | ventricular hemorrhage | | Yes | | None |
| 1. **20 DC in the validation cohort** | | | | | | | | |
| **1628912** | 41 | F | 5 | hypothalamic tumor | | None | | None |
| **2309485** | 42 | F | 5 | cerebellopontine angle tumor | | None | | None |
| **2149645** | 43 | F | 5 | cavernous hemangioma | | None | | None |
| **0918459** | 44 | F | 5 | frontal meningioma | | None | | None |
| **1429129** | 45 | F | 5 | thalamic tumor | | None | | None |
| **2419070** | 47 | F | 5 | Intraspinal tumor | | None | | None |
| **2601989** | 50 | F | 5 | ventricle tumor | | None | | None |
| **2808160** | 50 | F | 5 | pituitary tumor | | None | | None |
| **1841775** | 44 | F | 5 | intracranial metastases | | None | | None |
| **1844203** | 59 | F | 5 | meningioma | | None | | None |
| **1856905** | 57 | F | 5 | cerebellar tumor | | None | | None |
| **2769742** | 63 | F | 5 | obstructive hydrocephalus | | None | | None |
| **1400070** | 58 | F | 5 | hydrocephalus | | None | | None |
| **1561387** | 40 | M | 5 | hydrocephalus | | None | | None |
| **1621790** | 40 | M | 5 | cerebellar tumor | | Yes | | None |
| **2108094** | 42 | M | 5 | moyamoya disease | | None | | None |
| **2191457** | 61 | M | 5 | pituitary tumor | | None | | None |
| **2305380** | 53 | M | 5 | temporal glioma | | None | | None |
| **2319829** | 59 | M | 5 | cavernous hemangioma | | Yes | | None |
| **2244602** | 52 | M | 5 | arteriovenous malformation | | Yes | | None |

**HH:**Hunt and Hess grade**; FISHER:** Fisher grade**; GOS:** Glasgow outcome scale; **RIA**: ruptured intracranial aneurysm; **UIA**: unruptured intracranial aneurysm; **HC**: health controls; **DC:** disease controls.
