# Supplementary material for: Identification of the soluble form of tyrosine kinase receptor Axl as a potential biomarker for intracranial aneurysm rupture
Source: BMC Neurol. 2015 Mar 5;15:23. doi: 10.1186/s12883-015-0282-8 (PMC4375882; doi:10.1186/s12883-015-0282-8)
Supplement: Additional file 2: — The peptides identified in human CSF. [file 12883_2015_282_MOESM2_ESM.doc]

**Additional file 2. The peptides identified in human CSF**

| **Protein Name** | **IPI #** | **Peptides** | |
| --- | --- | --- | --- |
|  |  | **N*** | **Sequence** |
| 17-beta hydroxysteroid dehydrogenase | IPI00376206 | 2 | FAAVGFHR  GLTSELQALGK |
| 24 kDa protein | IPI00479531 | 17 | NEEYNK  TEDTIFLR  WFYIASAFR  SDVVYTDWK  TYMLAFDVNDEK  EQLGEFYEALDCLR  YVGGQEHFAHLLILR  NWGLSVYADKPETTK |
| 36 kDa protein | IPI00477751 | 3 | DSEYPFK  TGSTPYWIVR |
| 376 kDa protein | IPI00479143 | 5 | ELEAMR  TLELSEALR |
| 39 kDa protein | IPI00479497 | 2 | VVVNFAPTIQEIK  TMQVHLTVQVPPK |
| 56 kDa protein | IPI00479747 | 3 | TVFGTEPDMIR  LVPHMNVSAVEK |
| 66 kDa protein | IPI00472812 | 6 | VASYWLIK  ISELQNYQR  SRPLDYTFVPR  TFIVKPANGAMGHGISLIR |
| Acheron, isoform 1 | IPI00414661 | 2 | LFGTFGVISSVR  TTPVPLFPNENLPSK |
| Adrenomedullin 2 precursor | IPI00385109 | 2 | SPSSSLQPR  GAGLAPVMGQPLR |
| Afamin precursor | IPI00019943 | 4 | QDSISSK  FTFEYSR  RHPDLSIPELLR  IAPQLSTEELVSLGEK |
| Alcadein alpha-1 | IPI00007257 | 2 | GNLAGLTLR  AASEFESSEGVFLFPELR |
| Alpha-1-acid glycoprotein 2 precursor | IPI00020091 | 15 | NEEYNK  TEDTIFLR  WFYIASAFR  EHVAHLLFLR  SDVMYTDWK  TLMFGSYLDDEK |
| Alpha-1-antitrypsin precursor | IPI00305457 | 54 | IVDLVK  FLEDVK  FLENEDR  SPLFMGK  VVNPTQK  AVLTIDEK  FLENEDRR  QINDYVEK  SVLGQLGITK  FLEDVKK  LSSWVLLMK  LSITGTYDLK  LGMFNIQHCK  KQINDYVEK  KLSSWVLLMK  LVDKFLEDVK  GKWERPFEVK  ITPNLAEFAFSLYR  TDTSHHDQDHPTFNK  LQHLENELTHDIITK  VFSNGADLSGVTEEAPLK  DTEEEDFHVDQVTTVK  FNKPFVFLMIEQNTK  LYHSEAFTVNFGDTEEAK  ELDRDTVFALVNYIFFK  GTEAAGAMFLEAIPMSIP PEVK  LYHSEAFTVNFGDTEEAKK |
| Alpha-1B-glycoprotein precursor | IPI00022895 | 17 | ELLVPR  GVTFLLR  LLELTGPK  LETPDFQLFK  ATWSGAVLAGR  SGLSTGWTQLSK  CEGPIPDVTFELLR  TPGAAANLELIFVGPQHA GNYR  SWVPHTFESELSDPVELL VAES |
| Alpha-2-glycoprotein 1, zinc | IPI00166729 | 19 | IDVHWTR  SQPMGLWR  QDSQLQK  DYIEFNK  AGEVQEPELR  EDIFMETLK  WEAEPVYVQR  AYLEEECPATLR  AREDIFMETLK  QKWEAEPVYVQR  QDPPSVVVTSHQAPGEK  EIPAWVPFDPAAQITK  YYYDGKDYIEFNK  HVEDVPAFQALGSLNDL |
| Alpha-2-HS-glycoprotein precursor | IPI00022431 | 10 | VVHAAK  AHYDLR  ATLSEK  FSVVYAK  HTLNQIDEVK  TVVQPSVGAAAGPVVPP CPGR  HTFMGVVSLGSPSGEVS HPR |
| Alpha-2-macroglobulin precursor | IPI00478003 | 57 | VDSHFR  AFTNSK  SDIAPVAR  YGAATFTR  GEAFTLK  SLNEEAVK  QGIPFFGQVR  FEVQVTVPK  LPPNVVEESAR  SSGSLLNNAIK  AIGYLNTGYQR  VGFYESDVMGR  QTVSWAVTPK  LSFYYLIMAK  SIYKPGQTVK  HYDGSYSTFGER  NALFCLESAWK  NEDSLVFVQTDK  LVHVEEPHTETVR  DMYSFLEDMGLK  NQGNTWLTAFVLK  AAQVTIQSSGTFSSK  TAQEGDHGSHVYTK  ALLAYAFALAGNQDK  IAQWQSFQLEGGLK  TEHPFTVEEFVLPK  LLIYAVLPTGDVIGDSAK  QFSFPLSSEPFQGSYK  VDLSFSPSQSLPASHAHLR  LHTEAQIQEEGTVVELTGR  LLLQQVSLPELPGEYSMK  DLTGFPGPLNDQDDEDCINR  QQNAQGGFSSTQDTVVALHALSK  VVSMDENFHPLNELIPLVYIQDPK  AVDQSVLLMKPDAELSASSVYNLLPEK |
| AMBP protein precursor | IPI00022426 | 7 | ETLLQDFR  TVAACNLPIVR  AFIQLWAFDAVK  VVAQGVGIPEDSIFTMADR |
| Amyloid beta A4 precursor protein-binding family A member 1 | IPI00294556 | 4 | LHHYDER  MMQAQEAVSR |
| Amyloid-like protein 1 precursor | IPI00020012 | 21 | VLLALR  FLHQER  LVETHATR  VIALINDQR  MNPLEQYER  QMYPELQIAR  EWAMADNQSK  VEQATQAIPMER  HYQHVAAVDPEK  FQVHTHLQVIEER  GSTEQDAASPEKEK  AALEGFLAALQADPPQAER  QALNEHFQSILQTLEEQVSGER |
| Angiotensinogen precursor | IPI00032220 | 22 | VANPLSTA  LDTEDKLR  FMQAVTGWK  DPTFIPAPIQAK  AAMVGMLANFLGFR  SLDFTELDVAAEK  VLSALQAVQGLLVAQGR  QPFVQGLALYTPVVLPR  ADSQAQLLLSTVVGVFTAPGLHLK |
| Ankyrin repeat and FYVE domain protein 1 | IPI00159899 | 4 | SGCDVNSPR  EDFSSMSAQLLYK  LTPLHLAVQAGSEIIVR |
| Antithrombin III variant | IPI00032179 | 21 | ELFYK  LPGIVAEGR  LQPLDFK  IEDGFSLK  RVWELSK  SKLPGIVAEGR  EVPLNTIIFMGR  DIPMNPMCIYR  EVPLNTIIFMGR  TSDQIHFFFAK  KATEDEGSEQK  VAEGTQVLELPFK  FATTFYQHLADSK  EQLQDMGLVDLFSPEK  NDNDNIFLSPLSISTAFAMTK AFLEVNEEGSEAAASTAVVIAGR |
| Apolipoprotein A-I precursor | IPI00021841 | 55 | QLNLK  AHVDALR  DLEEVK  LAEYHAK  QKVEPLR  LSPLGEEMR  LHELQEK  LSPLGEEMR  AKPALEDLR  WQEEMELYR  THLAPYSDELR  ATEHLSTLSEK  QGLLPVLESFK  DLATVYVDVLK  VQPYLDDFQK  QKLHELQEK  THLAPYSDELR  ETEGLRQEMSK  VSFLSALEEYTK  DYVSQFEGSALGK  KWQEEMELYR  VKDLATVYVDVLK  LLDNWDSVTSTFSK  DSGRDYVSQFEGSALGK  EQLGPVTQEFWDNLEK  EQLGPVTQEFWDNLEKE TEGLR |
| Apolipoprotein A-II precursor | IPI00021854 | 6 | SYFEK  EQLTPLIK  SPELQAEAK  SKEQLTPLIK  VKSPELQAEAK |
| Apolipoprotein A-IV precursor | IPI00304273 | 27 | QLTPYAQR  LAPLAEDVR  IDQTVEELR  LEPYADQLR  ALVQQMEQLR  VNSFFSTFK  LTPYADEFK  ALVQQMEQLR  IDQNVEELK  TQVNTQAEQLR  LVPFATELHER  LLPHANEVSQK  SLAPYAQDTQEK  LGEVNTYAGDLQK  KLVPFATELHER  SELTQQLNALFQDK  SLAELGGHLDQQVEEFR |
| Apolipoprotein D precursor | IPI00006662 | 3 | VLNQELR  NILTSNNIDVK  CPNPPVQENFDVNK |
| Apolipoprotein E precursor | IPI00021842 | 69 | LASHLR  AQAWGER  WELALGR  ELQAAQAR  FWDYLR  DADDLQK  LAVYQAGAR  LGPLVEQGR  DRLDEVK  LQAEAFQAR  QWAGLVEK  ALMDETMK  LEEQAQQIR  LGADMEDVCGR  QQTEWQSGQR  AKLEEQAQQIR  AATVGSLAGQPLQER  SWFEPLVEDMQR  VQAAVGTSAAPVPSDNH  VEQAVETEPEPELR  GEVQAMLGQSTEELR  SELEEQLTPVAEETR  AYKSELEEQLTPVAEETR  WVQTLSEQVQEELLSSQVTQELR |
| ASRGL1 protein | IPI00169322 | 2 | AATVGYGILR  LTLFHIEQGK |
| AXL receptor tyrosine kinase, isoform 1 | IPI00296992 | 2 | APLQGTLLGYR  TTEATLNSLGISEELK |
| Baculoviral IAP repeat-containing protein 4 | IPI00303890 | 2 | NFPNSTNLPR  DSMQDESSQTSLQK |
| BAG-family molecular chaperone regulator-3 | IPI00000644 | 6 | VEAILEK  PQQPMTHR  SSLGSHQLPR |
| Beta-2-glycoprotein I precursor | IPI00298828 | 4 | VSFFCK  ATVVYQGER  KATVVYQGER |
| Beta-2-microglobulin precursor | IPI00004656 | 7 | IQVYSR  VNHVTLSQPK  VEHSDLSFSK |
| Biotinidase precursor | IPI00218413 | 4 | LSSGLVTAALYGR  VDLITFDTPFAGR  TSIYPFLDFMPSPQVVR |
| C4B1 | IPI00418163 | 88 | AINEK  ADLEK  VLQIEK  VEASISK  NVNFQK  GLESQTK  EFHLHLR  GQIVFMNR  VFALDQK  QGSFQGGFR  GLQDEDGYR  SHALQLNNR  VDFTLSSER  VEYGFQVK  VGDTLNLNLR  DKGQAGLQR  TYNVLDMK  LGQYASPTAK  DHAVDLIQK  AEFQDALEK  ITQVLHFTK  YVLPNFEVK  SHKPLNMGK  LNMGITDLQGLR  SFFPENWLWR  EMSGSPASGIPVK  AEMADQAAAWLTR  GSSTWLTAFVLK  KADGSYAAWLSR  GSFEFPVGDAVSK  DFALLSLQVPLK  KYVLPNFEVK  TEQWSTLPPETK  TTNIQGINLLFSSR  GLEEELQFSLGSK  EELVYELNPLDHR  HLVPGAPFLLQALVR  VLSLAQEQVGGSPEK  GPEVQLVAHSPWLK  GHLFLQTDQPIYNPGQR  AVGSGATFSHYYYMILSR  VGLSGMAIADVTLLSGFHALR  RGHLFLQTDQPIYNPGQR  ALEILQEEDLIDEDDIPVR  ASAGLLGAHAAAITAYALTLTK  VTASDPLDTLGSEGALSPGGVASLLR  TLEIPGNSDPNMIPDGDFNSYVR |
| Calreticulin precursor | IPI00020599 | 5 | PEDWDER  VHVIFNYK  IKDPDASKPEDWDER |
| Calsyntenin-1 precursor | IPI00413959 | 3 | LTVTAYDCGK  EGLDLQVLEDSGR  AASEFESSEGVFLFPELR |
| Carboxypeptidase E precursor | IPI00031121 | 2 | VAVPYSPAAGVDFELESFSER  KVAVPYSPAAGVDFELESFSER |
| Cathepsin D precursor | IPI00011229 | 6 | VGFAEAAR  FDGILGMAYPR  LVDQNIFSFYLSR  ISVNNVLPVFDNLMQQK |
| Cathepsin L precursor | IPI00012887 | 3 | LYGMNEEGWR  VFQEPLFYEAPR  NQGQCGSCWAFSATGALEGQMFR |
| CD59 glycoprotein precursor | IPI00011302 | 2 | ENELTYYCCK  FEHCNFNDVTTR |
| Cell surface glycoprotein MUC18 precursor | IPI00016334 | 4 | EETGQVLER  NGYPIPQVIWYK  GATLALTQVTPQDER |
| Chitinase-3 like protein 1 precursor | IPI00002147 | 11 | LVMGIPTFGR  TLLSVGGWNFGSQR  FSNTDYAVGYMLR  GNQWVGYDDQESVK  SFTLASSETGVGAPISGPGIPGR |
| Chromosome 1 open reading frame 27 | IPI00305788 | 2 | LWNFTEEEVSER  EIENGVYLINGQVK |
| Clusterin isoform 1 | IPI00400826 | 31 | ALQEYR  IDSLLENDR  FMETVAEK  KYNELLK  RPHFFFPK  TLLSNLEEAK  ELDESLQVAER  ASSIIDELFQDR  RELDESLQVAER  KTLLSNLEEAK  QQTHMLDVMQDHFSR  LFDSDPITVTVPVEVSR  EILSVDCSTNNPSQAK  EPQDTYHYLPFSLPHR  VTTVASHTSDSDVPSGVTEVVVK |
| Clusterin precursor | IPI00291262 | 19 | TLIEK  ALQEYR  IDSLLENDR  FMETVAEK  TLLSNLEEAK  ASSIIDELFQDR  LFDSDPITVTVPVEVSR |
| Coagulation factor XII precursor | IPI00019581 | 2 | EQPPSLTR  CFEPQLLR |
| Collagen alpha 1(I) chain precursor | IPI00297646 | 2 | GPAGPQGPR  STGGISVPGPMGPSGPR |
| Collagen alpha 1(VI) chain precursor | IPI00291136 | 4 | VPSYQALLR  VFSVAITPDHLEPR  ENYAELLEDAFLK  SLQWMAGGTFTGEALQYTR |
| Collagen alpha 2(I) chain precursor | IPI00304962 | 3 | GPAGPSGPAGK  GEAGAAGPAGPAGPR  GETGPSGPVGPAGAVGPR |
| Complement C1q subcomponent, C chain precursor | IPI00022394 | 3 | VVTFCGHTSK  FNAVLTNPQGDYDTSTGK |
| Complement C1s subcomponent precursor | IPI00017696 | 4 | LQVIFK  GFQVVVTLR  EPTMYVGSTSVQTSR  SNALDIIFQTDLTGQK |
| Complement C3 precursor | IPI00164623 | 16 | VVPEGIR  RQGALELIK  DFDFVPPVVR  SSLSVPYVIVPLK  AGDFLEANYMNLQR  VYAYYNLEESCTR  AGDFLEANYMNLQR  SYTVAIAGYALAQMGR  EGVQKEDIPPADLSDQVPDTESETR |
| Complement C5 precursor | IPI00032291 | 2 | VFQFLEK  TDAPDLPEENQAR |
| Complement component C6 precursor | IPI00009920 | 2 | GFVVAGPSR  TFSEWLESVK |
| Complement component C7 precursor | IPI00296608 | 5 | LTPLYELVK  SYTSHTNEIHK  LSGNVLSYTFQVK LIDQYGTHYLQSGSLGGEYR |
| Complement component C9 precursor | IPI00022395 | 8 | VVEESELAR  FTPTETNK  LSPIYNLVPVK  AIEDYINEFSVR  DVVLTTTFVDDIK |
| Complement factor I precursor | IPI00291867 | 3 | VFSLQWGEVK  AQLGDLPWQVAIK |
| Condensin subunit 2 | IPI00299507 | 3 | VFPMPLPR  EPCQVQSCQEEMISLGDGDIR  ATTLPTDFNYNVDTLVQLHLK |
| Contactin 2 precursor | IPI00024966 | 5 | GPPGPPGGVVVR  VTVTPDGTLIIR  FAQLNLAAEDTR  DIGDTTIQLSWSR  ETIGDLTILNAQLR WLLNEFPNFIPTDGR |
| Cortactin-binding protein 2 | IPI00103869 | 3 | PSTGSPLVSANAK  FNLNDPFLALQR  PVCTNPLSILEAVMAHCK |
| Corticosteroid-binding globulin precursor | IPI00027482 | 3 | MNTVIAALSR  GTWTQPFDLASTR |
| Cystatin C precursor | IPI00032293 | 7 | ASNDMYHSR  ALDFAVGEYNK  LVGGPMDASVEEEGVR  QIVAGVNYFLDVELGR |
| Death-associated protein kinase 1 | IPI00021250 | 3 | DKSGEMALHVAAR  YLCLMGASVEALTTDGK  NPLQVVLVATHADIMNVPR |
| Dickkopf related protein-3 precursor | IPI00002714 | 17 | DQDGEILLPR  SAVEEMEAEEAAAK  EVEELMEDTQHK  EVPDEYEVGSFMEEVR  LLDLITWELEPDGALDR  SLTEEMALGEPAAAAAALLGGEEI |
| DJ788L20.2 | IPI00100250 | 5 | INESLAQLK  LLGHLAACLR  AGFHECLAEVNR |
| DOC-2/DAB2 interactive protein | IPI00395467 | 7 | IASLDAANAR  LEEYETLFK  QGPSPVSPNALDR  MVIENDLSGLIDFTR |
| Dolichyl-P-Man:Man | IPI00012208 | 2 | FLQVNSAWR  FIWLSAFAIIVFR |
| Ephrin A1 isoform b precursor | IPI00377015 | 2 | LAADDPEVR  ITHSPQAHDNPQEK |
| Ephrin-B2 precursor | IPI00005126 | 2 | VGQDASSAGSTR  FQEFSPNLWGLEFQK |
| Epidermal growth factor receptor substrate 15 | IPI00292134 | 5 | YDEIFLK  SGLPDLILGK  QQVQELLDELDEQK  LNQQEQEDLELAIALSK |
| Extracellular matrix protein 1 | IPI00006969 | 3 | VTPNLMGHLCGNQR  CCDLPFPEQACCAEEEK |
| Extracellular superoxide dismutase [Cu-Zn] precursor | IPI00027827 | 5 | VTGVVLFR  VTEIWQEVMQR |
| FLJ00385 protein | IPI00168728 | 2 | SCDTPPPCPR  VVSVLTVLHQDWLNGK |
| Full-length cDNA clone CS0DN001YP04 of adult brain of Homo sapiens | IPI00383975 | 3 | LEGQEEEEDNR  PHTASCSAPGSAMR  PSPMPVSQECFETLR |
| Galectin-3 binding protein precursor | IPI00023673 | 17 | VEIFYR  AVDTWSWGER  KSQLVYQSR  ASHEEVEGLVEK  SDLAVPSELALLK  ELSEALGQIFDSQR  YSSDYFQAPSDYR  TLQALEFHTVPFQLLAR  IYTSPTWSAFVTDSSWSAR  YYPYQSFQTPQHPSFLFQDK |
| Gamma-taxilin | IPI00019994 | 3 | ALGAHLEAEPK  FEEFQTTMAK  TDPPDGQQDSECNR |
| Gelsolin precursor | IPI00026314 | 2 | EGGQTAPASTR  YIETDPANR |
| Glucocorticoid receptor DNA binding factor 1 isoform b | IPI00477498 | 2 | MQASPEYQDYVYLEGTQK  IPFDLMDTVPAEQLYEAHLEK |
| Glutamate decarboxylase, 65 kDa isoform | IPI00012796 | 2 | GAAALGIGTDSVILIK  PTLAFLQDVMNILLQYVVK |
| Grb10 interacting GYF protein 1 | IPI00428657 | 2 | QQELLLK  SIEEGDGAFGR |
| Guanylate binding protein 4 | IPI00419541 | 2 | LSEHLTESILR  AADHYSQQMAQQLR |
| H2AFY protein | IPI00304171 | 4 | QTAAQLILK  SIAFPSIGSGR  HILLAVANDEELNQLLK |
| Haptoglobin precursor | IPI00478493 | 20 | DYAEVGR  FTDHLK  VGYVSGWGR  QLVEIEK  GSFPWQAK  ILGGHLDAK  HYEGSTVPEK  VTSIQDWVQK  DIAPTLTLYVGK  HYEGSTVPEKK  TEGDGVYTLNNEK  YVMLPVADQDQCIR  SPVGVQPILNEHTFCAGMSK |
| Hemoglobin gamma-G | IPI00464992 | 2 | LLVVYPWTQR  GTFAQLSELHCDK |
| Hemopexin precursor | IPI00022488 | 33 | LHIMAGR  LWWLDLK  DYFMPCPGR  VDGALCMEK  VWVYPPEK  NFPSPVDAAFR  RLWWLDLK  GGYTLVSGYPK  VWVYPPEKK  YYCFQGNQFLR  EWFWDLATGTMK  LYLVQGTQVYVFLTK  SGAQATWTELPWPHEK  LLQDEFPGIPSPLDAAVECHR  EVGTPHGIILDSVDAAFICPGSSR |
| Hepatoma-derived growth factor-related protein 2, isoform 2 | IPI00013290 | 5 | GYPHWPAR  TSDQDFTPEK  LAGEELAGEEAPQEK |
| HMG-box containing protein | IPI00300222 | 4 | NTMEPVHK  PPDFISISASK  VQLLGADGLEQDVGETEDDESPEQR |
| Hypothetical protein | IPI00026195 | 7 | FSGSGSGTDFTLK  SGTASVVCLLNNFYPR  VYACEVTHQGLSSPVTK  TVAAPSVFIFPPSDEQLK  VDNALQSGNSQESVTEQDSK |
| Hypothetical protein | IPI00384355 | 2 | LTVLGQPK  AGVETTTPSK |
| Hypothetical protein | IPI00395435 | 2 | PGQAPVLVIYK  SYELTQPPSVSVSPGQTAR |
| Hypothetical protein | IPI00430804 | 2 | YAASSYLSLTPEQWK  ATLVCLISDFYPGAVTVAWK |
| Hypothetical protein DKFZp313D1622 | IPI00296554 | 6 | PLECSCFR  TFDVEHSHVR  TSIWDETLYK  NFAEIIEADEVLLFER |
| Hypothetical protein DKFZp434C011 | IPI00152946 | 2 | DLLMK  LSTIDESGSILSDISFDK |
| Hypothetical protein DKFZp434O194 | IPI00031282 | 4 | ILAVITIQAGVR  TPSLAHLDTCLSK  CFQSCQAHACSVCHSLSSR |
| Hypothetical protein DKFZp686A05192 | IPI00375705 | 3 | VPPTLEVTQQPVR  ISNITPADAGTYYCVK |
| Hypothetical protein DKFZp686C15213 | IPI00426051 | 17 | DTLMISR  GLPAPIEK  VDKTVER  GPSVFPLAPCSR  EPQVYTLPPSR  NSLYLQMNSLR  EPQVYTLPPSREEMTK  TTPPMLDSDGSFFLYSK |
| Hypothetical protein DKFZp686D0623 | IPI00470584 | 2 | LSPDIMK  VLGELNILPNTQK |
| Hypothetical protein DKFZp686G11190 | IPI00426007 | 5 | EEQYNSTYR  EPQVYTLPPSR  FNWYVDGVEVHNAK |
| Hypothetical protein DKFZp686G21220 | IPI00423460 | 6 | YLTWASR  SAVQGPPER  DTSTNIVYMEMR  DASGVTFTWTPSSGK |
| Hypothetical protein DKFZp686I04196 | IPI00399007 | 3 | DTLMISR  EPQVYTLPPSR  STSESTAALGCLVK |
| Hypothetical protein DKFZp686I15212 | IPI00418153 | 15 | DTLMISR  KPGASVK  ALPAPIEK  GPSVFPLAPCSR  EPQVYTLPPSR  WYVDGVEVHNAK  EPQVYTLPPSREEMTK |
| Hypothetical protein DKFZp761H2017 | IPI00166552 | 9 | IQVWPSR  NPEPPSGVR  DPGDYCFSIR  FHWIHTPLVLTGK  DNGSLALPADAHLYR |
| Hypothetical protein FLJ11594 | IPI00386962 | 8 | GPVPAIHK  PGSVLLLK  GTIQSSPQNR  TPNSSCSTPSR  DPTGEMQGTVHR  DPSCFLCTYSIVMVLR |
| Hypothetical protein FLJ11633 | IPI00386957 | 2 | TVMLFNLGSAYCLR  NSNQLGGNTESSESSETCSSK |
| Hypothetical protein FLJ20475 | IPI00183065 | 2 | PLHSLSVLAFDQER  GINLPTPPGLSPGDLDVFVR |
| Hypothetical protein FLJ22037 | IPI00025094 | 5 | ALEELHQK  AEIDDLNASMETIQK  HVDSMAELTEHVESLQR |
| Hypothetical protein FLJ23322 | IPI00166509 | 2 | CLGWNFDFSIFNK  SVGHTEDAWQSNFLHPVIYYYR |
| Hypothetical protein FLJ23757 | IPI00154836 | 2 | SFLELEAPPLPQPR  STQIPQWCAEFALAR |
| Hypothetical protein FLJ25298 | IPI00386524 | 5 | ASANMFR  SYAFTWVR  DLCGCYSVSSVLPGCAEPWNHGK |
| Hypothetical protein FLJ34458 | IPI00300753 | 2 | LAPNDHLLYSNR  DNLELPHCSSQEEAAAR |
| Hypothetical protein FLJ35721 | IPI00167940 | 2 | EETGIICPER  CQEAIVSPQAR |
| Hypothetical protein FLJ41598 | IPI00419164 | 2 | ELMYK  QHNAYGGFSSTQDTVVALQALAK |
| Hypothetical protein FLJ42206 | IPI00446339 | 6 | LEHLQEK  LIVEGHLTK  DDLQGAQSEIEAK  IEALQADNDFTNER |
| Hypothetical protein FLJ45525 | IPI00299571 | 9 | LTPEWK  VGAVDADK  PEDYQGGR  GESPVDYDGGR |
| Hypothetical protein FLJ46072 | IPI00394829 | 5 | LNPLVQR  SDSLGTQGR  SSPVPPVPER |
| Hypothetical protein FLJ90661 | IPI00168352 | 6 | AERVAR  PEPSAR  EEVGSCWNDSR |
| Hypothetical protein KIAA0156 | IPI00006025 | 2 | EELEEICK  PMFVSPCVDK |
| Hypothetical protein KIAA0792 | IPI00477163 | 7 | TSSEASIR  NLSIQGLR  LPGLILYTFR  WTVTFAADPEDICWK |
| Hypothetical protein MGC10992 | IPI00059169 | 2 | GSLLTILGSPSPER  LPWEDTAATEEEASK |
| Hypothetical protein pp6318 | IPI00103828 | 4 | SWLMSGK  TCHESYAR  TEAGFSGIQDVYSSTPNHDNK |
| Ig kappa chain V-III region WOL | IPI00387118 | 8 | TFGQGTK  LLIYGASSR  FSGSGSGTDFTLTISR  EIVLTQSPGTLSLSPGER |
| Ig kappa chain V-IV region B17 precursor | IPI00386133 | 5 | PGQPPK  ESGVPDR  LLIYWASTR |
| Immunoglobulin-like domain protein MGC33530 precursor | IPI00290411 | 6 | FTEFPR  DPDSDGTK  VQGNDISHK  VTDANYGELQEHK |
| Importin 9 | IPI00185146 | 2 | AAAEEQIK  ELLPNGLR |
| Insulin-like growth factor binding protein 7 precursor | IPI00016915 | 2 | AITQVSK  ITVVDALHEIPVK |
| Inter-alpha-trypsin inhibitor heavy chain H1 precursor | IPI00292530 | 6 | AAISGENAGLVR  QYYEGSEIVVAGR  GSLVQASEANLQAAQDFVR  FAHYVVTSQVVNTANEAR |
| Inter-alpha-trypsin inhibitor heavy chain H2 precursor | IPI00305461 | 2 | ETAVDGELVVLYDVK  NILFVIDVSGSMWGVK |
| Isg20 protein | IPI00031824 | 3 | LVVGHDLK  DDTFEAQGGLIICPR |
| ISLR precursor | IPI00023648 | 2 | ALPGTPVASSQPR  EVPLLQSLWLAHNEIR |
| JRK protein | IPI00216621 | 2 | ALEQR  FFASSDSNK |
| Keratin 1 | IPI00220327 | 3 | QISNLQQSISDAEQR  NKLNDLEDALQQAK  THNLEPYFESFINNLR |
| Keratin 9 | IPI00019359 | 24 | KAALEK  LASYLDK  TLLDIDNTR  FEMEQNLR  KGPAAIQK  IQDWYDK  FSSSSGYGGGSSR  QEYEQLIAK  QVLDNLTMEK  IQDWYDKK  VQALEEANNDLENK  GGSGGSYGGGGSGGGY GGGSGSR  HGVQELEIELQSQLSK  SDLEMQYETLQEELMALK  EIETYHNLLEGGQEDFESSGAGK  GGGGSFGYSYGGGSGGGFSASSLGGGFGGGSR |
| KIAA0319 protein | IPI00006524 | 2 | GSPSGIWGDSPEDIR  NYSPYYNTIDDLKDQIVD LTVGNNK |
| KIAA0690 | IPI00101186 | 3 | LPSGVSAKLKR  TLGMAISERPDLR  IRKAAQHGVCSVLK |
| Laminin alpha-1 chain precursor | IPI00375294 | 2 | KQAASIK  ETHQLLQDSTMATLLAGRKVK |
| Leucine-rich alpha-2-glycoprotein precursor | IPI00022417 | 3 | VAAGAFQGLR  DLLLPQPDLR  TLDLGENQLETLPPDLLR |
| Limbic system-associated membrane protein precursor | IPI00013303 | 5 | SGIIFAGHDK  AANEVSSADVK  VTVNYPPTITESK  EFEGEEEYLEILGITR |
| LP2209 | IPI00428724 | 2 | ETEWDLR  RAAQHTVGGTK |
| Lumican precursor | IPI00020986 | 3 | SLEYLDLSFNQIAR  ISETSLPPDMYECLR  LPSGLPVSLLTLYLDNNK |
| Lysosomal-associated membrane protein 2C | IPI00216172 | 5 | LNSSTIK  IPLNDLFR  VQPFNVTQGK  IAVQFGPGFSWIANFTK |
| Lysosomal-associated multitransmembrane protein | IPI00013827 | 2 | VYMFK  QTCCCFNVR |
| Metalloproteinase inhibitor 1 precursor | IPI00032292 | 4 | SEEFLIAGK  GFQALGDAADIR  FVYTPAMESVCGYFHR |
| Microtubule associated serine/threonine kinase-like | IPI00074258 | 6 | SCVNLAKK  DTTPYSSK  CLTSNLLQSR  DYLSSSFLCSDDDR |
| Mimecan precursor | IPI00025465 | 8 | KLTAK  RLPIGSYF  ESAYLYAR  DFADIPNLR  LEGNPIVLGK  LSLLEELSLAENQLLK  LDFTGNLIEDIEDGTFSK |
| Monocyte differentiation antigen CD14 precursor | IPI00029260 | 11 | ELTLEDLK  FPAIQNLALR  LKELTLEDLK  LTVGAAQVPAQLLVGALR  AFPALTSLDLSDNPGLGER  VLSIAQAHSPAFSCEQVR  ITGTMPPLPLEATGLALSSLR |
| MUF1 protein | IPI00397576 | 3 | ELHPQPPPMR  HLLFSDVAAQQSLR  CAAALMASRRKSEAK |
| Mutated in bladder cancer 1 | IPI00168663 | 8 | QAKEDEK  FQERDLHK  TQDEVQQHEK  TKKIGPTGSGPLLHIPHR  LAKLKEKVENNVSRDPSR  LLLESYTQQKKEQEEFLR |
| Myosin heavy chain, cardiac muscle alpha isoform | IPI00302328 | 9 | EAEFQK  QLEEEGK  GGKKQLQK  ELEEISER  QLEEKEALISQLTR  TEELEEAKKKLAQR |
| N-acetylgalactosamine-4-O-sulfotransferase | IPI00300838 | 2 | NLPAPDQPQPPLQR  VLYCEVPKAGCSNWK |
| N-acetyllactosaminide beta-1,3-N-acetylglucosaminyltransferase | IPI00009997 | 12 | VPTFDER  YEAAVPDPR  EPGEFALLR  TALASGGVLDASGDYR  WEGPLSVSVFAATK  EMLDQSNQWGGTALVVPAFEIR |
| N-acetyltransferase 5 isoform c | IPI00375483 | 2 | QLGYSVYR  VSNQVAVNMYK |
| Nebulin | IPI00303335 | 3 | AGELLSDTIYR  FSSPVDMLSILLAKK  TSIHVMPDTPDILLAK |
| Nectin-like protein 2 | IPI00003813 | 3 | CEASNIVGK  NLMIDIQK  SDDSVIQLLNPNR |
| Neural cell adhesion molecule | IPI00299059 | 9 | VIAVNEVGR  GYQINWWK  TTVILPLAPFVR  GNPEPTFSWTK  GDLYFANVEEK  VMTPAVYAPYDVK  KTTVILPLAPFVR |
| Neural cell adhesion molecule 1, 140 kDa isoform precursor | IPI00435020 | 3 | GLGEISAASEFK  TQPVQGEPSAPK  FFLCQVAGDAK |
| Neural cell adhesion molecule 2 | IPI00478109 | 6 | IEIFQTLPVR  ASGSPEPAISWFR  MILEIAPTSDNDFGR  LTIYNANIEDAGIYR |
| Neuroblastoma suppressor of tumorigenicity 1 precursor | IPI00013299 | 2 | LALFPDK  SAWCEAK |
| Neurocan core protein precursor | IPI00159927 | 3 | YPIQTPR  ELGGEVFYVGPAR  DFQWTDNTGLQFENWR |
| Neuronal pentraxin I precursor | IPI00220562 | 2 | WTFEACR  FQLTFPLR |
| Neurosecretory protein VGF precursor | IPI00289501 | 10 | FQLTFPLR  GLQEAAEER  ASWGEFQAR  VNLESPGPER  LLQQGLAQVEAGR  APLPPPAPSQFQAR  TPAAETLSQLGQTLQSLK |
| NICE-4 protein | IPI00005416 | 2 | NPSDSAVHSPFTK  STQTRRYPSSISSSPQK |
| NifU-like protein HIRIP5 | IPI00455153 | 2 | FIPGKPVLETR  TMDFPTPAAAFR |
| Nogo receptor-like 3 | IPI00328746 | 2 | LFLQNNLIR  SLEPDTFQGLER |
| Nucleolysin TIAR | IPI00005615 | 3 | STQENNTK  FEDVVNQSSPK  QTFSPFGQIMEIR |
| Obscurin | IPI00479915 | 10 | HLPLDEPAELGLR  YLPFEFMIFRKVPK  GFLRPSASLPEEAEASER  LPSAPSGGAPIRDMGHPQGSK  GRPEGLEKEGPPRKKPGLASFR |
| Opioid binding protein/cell adhesion molecule precursor | IPI00001662 | 6 | LATGLDGMR  STILYAGNDK  LLFLVPTGVPVR  GILSCEASAVPMAEFQWFK |
| OTTHUMP00000022089 | IPI00170641 | 5 | LDLGGSER  DPFEDLLQK  EQPGTFDYQR  ESDSAEGDEAESPEQQVR |
| P protein | IPI00028627 | 2 | LLLGKVLALEHLLARR  ASLQQTQAVPLLMAHQYLR |
| Peroxisomal targeting signal 1 receptor | IPI00032931 | 5 | LANSEFLK  QACETLRDWLR  QIGEGQVSLESGAGSGR |
| Phosphatidylcholine-sterol acyltransferase precursor | IPI00022331 | 4 | SSGLVSNAPGVQIR  LEPGQQEEYYR  ITTTSPWMFPSR |
| Pigment epithelium-derived factor precursor | IPI00006114 | 4 | VPMMSDPK  LDLQEINNWVQAQMK  EIPDEISILLLGVAHFK  ALYYDLISSPDIHGTYK |
| Plasma kallikrein precursor | IPI00008558 | 11 | LSMDGSPTR  GEIQNILQK  QCGHQISACHR  FGCFLKDSVTGTLPK  LCNTGDNSVCTTKTSTR  GGDVASMYTPNAQYCQMR |
| Plasma protease C1 inhibitor precursor | IPI00291866 | 17 | FPVFMGR  TLYSSSPR  LLDSLPSDTR  FQPTLLTLPR  TNLESILSYPK  LVLLNAIYLSAK  VTTSQDMLSIMEK  LEDMEQALSPSVFK  GVTSVSQIFHSPDLAIR |
| Plasminogen precursor | IPI00019580 | 10 | LFLEPTR  DVVLFEK  WELCDIPR  WEYCNLK  FVTWIEGVMR  EAQLPVIENK  HSIFTPETNPR  VILGAHQEVNLEPHVQEI EVSR |
| PREDICTED: chromosome 20 open reading frame 142 | IPI00374076 | 8 | AGLVLR  KEHQSK  CEWLLR  NVLNVYFVK |
| PREDICTED: dynein, cytoplasmic, heavy polypeptide 2 | IPI00171494 | 2 | QISKERANYFK  LQNLLSELEAGLGIVLR |
| PREDICTED: hypothetical protein XP_291007 | IPI00216817 | 2 | LVHTTPLPEEMNLQR  QKELEKCKGDLQSTDVEK |
| PREDICTED: KIAA0146 protein | IPI00029021 | 7 | DQPCEEIK  LYQPPVTR  HQCISYQK  YHVQFASDAR  QLEGKSCSLVGMK |
| PREDICTED: KIAA1509 | IPI00029170 | 2 | ELLLQEDDSGSDTK  FDELKEQHQTMDISLTK |
| PREDICTED: KIAA1522 protein | IPI00001632 | 2 | GGWDHGDTQSIQSSR  EDVGAPLVTPSLLQMVR |
| PREDICTED: odz, odd Oz/ten-m homolog 3 | IPI00398020 | 4 | TDAYNQK  CLCFSGWK  CAEHGTCKDGK |
| PREDICTED: similar to RIKEN cDNA 1700022C21 | IPI00400925 | 2 | HSSPCAICVPLEK  ALGNHQPLPYIER |
| PREDICTED: similar to TAR DNA binding protein | IPI00147770 | 3 | TGHSKGFGFVR  CTEDMTEDELR  TSDLIVLGLPWK |
| PREDICTED: similar to tumor necrosis factor, alpha-induced protein 2 | IPI00073442 | 3 | ALTQVSK  QLLAAFEQLLR  AAGAISAELEATTLR |
| PRF1 protein | IPI00293423 | 3 | FVPGAWLAGEGVDVTSLR  ALGDLPHHFNASTQPAYLR  FYSFHVVHTPPLHPDFKR |
| Prion protein | IPI00382843 | 2 | QHTVTTTTK  VVEQMCITQYER |
| Procollagen C-proteinase enhancer protein precursor | IPI00299738 | 7 | GFLLWYSGR  GVSYLLMGQVEENR  YDALEVFAGSGTSGQR |
| Progesterone-induced blocking factor 1 | IPI00472584 | 2 | TNQEIDQLRNASR  QSVHLARRVLQLEK |
| Prostaglandin-H2 D-isomerase precursor | IPI00013179 | 32 | GPGEDFR  MATLYSR  FTAFCK  AELKEK  KAALSMCK  WFSAGLASNSSWLR  TMLLQPAGSLGSYSYR  SVVAPATDGGLNLTSTFLR  AQGFTEDTIVFLPQTDK  SPHWGSTYSVSVVETDYDQYALLYSQGSK |
| Protein F25965 | IPI00062869 | 8 | SNTYVIK  SHMDRER  AWMRNSPSVR  LPPPTPPGPPGDACRSR |
| Protein kinase C-binding protein NELL2 precursor | IPI00015260 | 8 | TYFEGER  AFLFQDTPR  IMELQDILAK  ASTATAEQFFQK  VVEKPSTDLPLGTTFWLGQR |
| Protein tyrosine phosphatase, non-receptor type substrate 1 precursor | IPI00332887 | 4 | SVLVAAGETATLR  TETASTVTENK  AKPSAPVVSGPAAR  VPPTLEVTQQPVR |
| Prothrombin precursor | IPI00019568 | 9 | VIDQFGE  YGFYTHVFR  ELLESYIDGR  TATSEYQTFFNPR  SEGSSVNLSPPLEQCVPDR  IVEGSDAEIGMSPWQVMLFR |
| PTPL1-associated RhoGAP | IPI00152011 | 4 | EILAQLR  LEEEALQK  LVEFLITYSQK |
| Receptor-interacting serine/threonine-protein kinase 2 | IPI00021917 | 2 | LHHCPGNHSWDSTISGSQR EDIVNQMTEACLNQSLDALLSRDLIMK |
| Retinoblastoma-associated factor 600 | IPI00180305 | 4 | FVPLILAR  LDSVACDVLFSK  YDVEIVEEYFAR  LLTSLFQDLQVEALHK |
| RGD, leucine-rich repeat, tropomodulin and proline-rich containing protein | IPI00456628 | 2 | GGLGPPAGVANSLPPQLFAAVSR LPPDALRALLDGLALNTHLRDLHLDLSACELR |
| Ribonuclease pancreatic precursor | IPI00014048 | 5 | NGQGNCYK  YPNCAYR  SNSSMHITDCR |
| SEC14 and spectrin domains 1 | IPI00329002 | 9 | SSGDTLPR  QASQLEFR  SVDVGLQGLR  SLQQQLSDVCYR  QFTIASEERVHR  FVDVAQSTYDYGR |
| Secretogranin I precursor | IPI00006601 | 24 | KEELVAR  KQASAIK  NYPSLELDK  SSQGGSLPSEEK  DPADASEAHESSSR  ASEEEPEYGEEIK  GEAGAPGEEDIQGPTK  GYPGVQAPEDLEWER  EDEEEEEGENYQK  LLRDPADASEAHESSSR  HLEEPGETQNAFLNER  APRPQSEESWDEEDKR  SQREDEEEEEGENYQK  SSQESGEEAGSQENHPQESK  ADEPQWSLYPSDSQVSEEVK |
| Secretogranin II precursor | IPI00009362 | 5 | IESQTQEEVR  LYTDDEDDIYK  ANNIAYEDVVGGEDWNPVEEK |
| Secretogranin III precursor | IPI00292071 | 3 | NIEWLK  QADAYVEK  YGTISPEEGVSYLENLDEMIALQTK |
| Selenoprotein S | IPI00020468 | 3 | AAAAVEPDVVVK  MQEELNAQVEK  QLEEEKRRQK |
| Semaphorin 7A precursor | IPI00025257 | 5 | IFAVWK  VYLFDFPEGK  AAAIQTMSLDAER  LQDVFLLPDPSGQWR  VVEPGEQEHSFAFNIMEIQPFR |
| Septin 10 isoform 2 | IPI00412153 | 2 | VNIIPVIAKADTVSK  VNAAMNGQLPFAVVGSMDEVK |
| Serine (or cysteine) proteinase inhibitor, clade A (alpha-1 antiproteinase, antitrypsin | IPI00396348 | 5 | YLPCSVLR  WADLSGITK  LGFTDLFSK  DFYVDENTTVR |
| Serotransferrin precursor | IPI00022463 | 59 | DLLFK  SCHTAVGR  APNHAVVTR  NPDPWAK  ASYLDCIR  DSAHGFLK  KDSSLCK  DGAGDVAFVK  YLGEEYVK  KPVDEYK  DSGFQMNQLR  DKEACVHK  EGYYGYTGAFR  HQTVPQNTGGK  DLLFRDDTVCLAK  FDEFFSEGCAPGSK  KPVEEYANCHLAR  TAGWNIPMGLLYNK  EDPQTFYYAVAVVK  SKEFQLFSSPHGK  ADRDQYELLCLDNTR  NLNEKDYELLCLDGTR  SAGWNIPIGLLYCDLPEPR  SDNCEDTPEAGYFAVAVVK  EDLIWELLNQAQEHFGK  SMGGKEDLIWELLNQAQEHFGK  KPVDEYKDCHLAQVPSHTVVAR |
| SERPINC1 protein | IPI00165421 | 12 | RVWELSK  EVPLNTIIFMGR  DIPMNPMCIYR  TSDQIHFFFAK  KATEDEGSEQK  FATTFYQHLADSK  ATEDEGSEQKIPEATNR  AFLEVNEEGSEAAASTAVVIAGR  NDNDNIFLSPLSISTAFAMTK |
| SERPIND1 protein | IPI00292950 | 4 | NFGYTLR  TLEAQLTPR  YEITTIHNLFR |
| Serum albumin precursor | IPI00022434 | 4 | LDELRDEGK  TYETTLEK  SHCIAEVENDEMPADLPS LAADFVESK |
| SEZ6L2 protein | IPI00306470 | 3 | HHYQAGESLR  TASDAGFPVGSHVQYR  LHLHFERVSLDEDNDRLMVR |
| Similar to peptide N-glycanase homolog | IPI00165496 | 5 | ISGSVAWR  IGNTAFSTR  SLLPSDDELK  FECGSVGLKVDSISIR |
| Small intestine SPAK-like kinase | IPI00457335 | 2 | IVDDPKALK  AKKVRRVPGSSGHLHK |
| SPARC-like protein 1 precursor | IPI00296777 | 5 | FFEECDPNK  VHAVDSCMSFQCK  TGLEAISNHKETEEK  NILMQLYEANSEHAGYLNEK |
| Spectrin beta chain, brain 4 | IPI00219168 | 3 | HQDLEKLLAAQEEK  GQWLAQAAQGHAFLGR  TEMEQELLLQPQELKPGR |
| Splice isoform 1 of adenomatous polyposis coli protein | IPI00012391 | 8 | EGSVSSR  LETEASNMK  HETGSHDAER  IQQIEKDILR |
| Splice isoform 1 of amine oxidase flavin containing domain protein 2 | IPI00456631 | 2 | GIFGSSAVPQPKETVVSR  DLTALCKEYDELAETQGK |
| Splice isoform 1 of B-lymphocyte antigen precursor | IPI00024024 | 4 | VVPEDGSPEKR  ATGDGLADRHKR  HAYKDDSPRRRSTSPDHTR |
| Splice isoform 1 of brevican core protein precursor | IPI00456623 | 8 | WTFLSR  GVVFLYR  YPIQTPR  YPIVTPSQR  GAIYSIPIMEDGGGGSSTPEDPAEAPR |
| Splice isoform 1 of complement factor B precursor | IPI00019591 | 16 | KVGSQYR  DISEVVTPR  ISVIRPSK  LQDEDLGFL  KEVYIK  VASYGVKPR  DAQYAPGYDK  EELLPAQDIK  VKDISEVVTPR  YGLVTYATYPK  KDNEQHVFK  EKLQDEDLGFL  QLNEINYEDHK  EAGIPEFYDYDVALIK |
| Splice isoform 1 of complement factor H precursor | IPI00029739 | 8 | KFVQGK  IDVHLVPDR  EFDHNSNIR  GEWVALNPLR  SSQESYAHGTK  EIMENYNIALR  IVSSAMEPDREYHFGQAVR |
| Splice isoform 1 of contactin 1 precursor | IPI00029751 | 2 | HSIEVPIPR  AHSDGGDGVVSQVK |
| Splice isoform 1 of COP9 signalosome complex subunit 1 | IPI00479323 | 8 | DIIFK  CYSRAR  NVISSSSFK  AESTPEIAEQR |
| Splice isoform 1 of ecto-ADP-ribosyltransferase 3 precursor | IPI00013682 | 2 | TQIFLPMNFK  EDYIYGFQFKAFHFYLTR |
| Splice isoform 1 of EGF-containing fibulin-like extracellular matrix protein 1 precursor | IPI00029658 | 3 | NPCQDPYILTPENR  EHIVDLEMLTVSSIGTFR  SVPSDIFQIQATTIYANTINTFR |
| Splice isoform 1 of erythrocyte membrane protein band 4.2 | IPI00028614 | 2 | CCEDGTLELTDSNTK  LFVRRGQPFTIILYFR |
| Splice isoform 1 of fibrinogen gamma chain precursor | IPI00021891 | 3 | NWIQYK  TSTADYAMFK  LTIGEGQQHHLGGAK |
| Splice isoform 1 of fibulin-1 precursor | IPI00296534 | 3 | TGYYFDGISR  IIEVEEEQEDPYLNDR  AITPPHPASQANIIFDITEGNLR |
| Splice isoform 1 of high-affinity cGMP-specific 3',5'-cyclic phosphodiesterase 9A | IPI00008806 | 2 | QGMITLILATDMAR  SEGLPVAPFMDRDKVTK |
| Splice isoform 1 of inter-alpha-trypsin inhibitor heavy chain H4 precursor | IPI00294193 | 13 | LGVYELLLK  YIFHNFMER  VTIGLLFWDGR  ETLFSVMPGLK  AGFSWIEVTFK  AEAQAQYSAAVAK  ITFELVYEELLK  SPEQQETVLDGNLIIR  QGPVNLLSDPEQGVEVTGQYER |
| Splice isoform 1 of neogenin precursor | IPI00023814 | 3 | GHESEDSMSTLAGRRGMR  GRRSSTWSMTAHGTTFELVPTSPPK  NANATTLSYLVTGLKPNTLYEFSVMVTK |
| Splice isoform 1 of proactivator polypeptide precursor | IPI00012503 | 6 | LIDNNK  TASDCGAVK  TEKEILDAFDK  NVIPALELVEPIKK |
| Splice isoform 1 of serologically defined colon cancer antigen 1 | IPI00301618 | 8 | AIQVVR  ALQQEK  AAEPLLTLER  DQQQNEIIVK  QLGVDRIVDFQFGSDEAAYHLIIELYDR |
| Splice isoform 1 of tetratricopeptide repeat protein 7A | IPI00397195 | 2 | IKDSMPLLEK  DGSFGEGLTMK |
| Splice isoform 1 of ubiquitin carboxyl-terminal hydrolase 6 | IPI00423562 | 5 | NSSPNSSPR  FQFVNDQWIK  CYGDLVQELWSGTQK  HLRASTKKLTRKQGDLPPPAKR |
| Splice isoform 2 of adenosine kinase | IPI00234368 | 2 | VMPYVDILFGNETEAATFAR  ENILFGMGNPLLDISAVVDKDFLDK |
| splice isoform 2 of contactin 1 precursor | IPI00216641 | 20 | GPPGPPGGLR  VVATNTLGR  ENIHYQR  IVESYQIR  ASPFPVYK  VTVTNPDTGR  FIPLIPIPER  VQVTSQEYSAR  ELTITWAPLSR  TDPPIIEGNMEAAR  TDGAAPNVAPSDVGGGGGR  AVDLIPWMEYEFR  YWAAHDKEEAANR  WLLNEFPVFITMDK  STEATLSFGYLDPFPPEERPEVR |
| Splice isoform 2 of ectonucleotide pyrophosphatase/phosphodiesterase 2 | IPI00303210 | 10 | VWNYFQR  WVEELMK  IVGQLMDGLK  RIEDIHLLVER  WWGGQPLWITATK  QMSYGFLFPPYLSSSPEAK  TEFLSNYLTNVDDITLVPGTLGR |
| Splice isoform 2 of fibrinogen alpha/alpha-E chain precursor | IPI00029717 | 4 | TVIGPDGHK  QFTSSTSYNR  NPSSAGSWNSGSSGPG STGNR  TFPGFFSPMLGEFVSETESR |
| Splice isoform 2 of interleukin-17E precursor | IPI00332192 | 2 | WSTVPVPPLEPARPNR  GNSELLYHNQTVFYRRPCHGEK |
| Splice isoform 2 of kininogen precursor | IPI00215894 | 15 | VQVVAGK  EETTSHLR  QVVAGLNFR  YFIDFVAR  KYFIDFVAR  TVGSDTFYSFK  IGEIKEETTSHLR  YNSQNQSNNQFVLYR  DIPTNSPELEETLTHTITK |
| Splice isoform 2 of neuronal-specific septin 3 | IPI00384187 | 3 | TVEIKAIGHVIEEGGVK  VVNIIPVIAKADTMTLEEK  KELEVNGIEFYPQKEFDEDLEDK |
| Splice isoform 2 of phospholipid transfer protein precursor | IPI00217778 | 2 | IYSNHSALESLALIPLQAPLK  FRRQLLYWFFYDGGYINASAEGVSIR |
| Splice isoform 2 of poliovirus receptor related protein 1 precursor | IPI00218887 | 4 | HVYGNGYSK  NPNGTVTVISR  GPINYSLAGTYICEATNPIGTR |
| Splice isoform 2 of serine/threonine-protein kinase RIPK4 | IPI00215935 | 9 | NGHLATVK  LESEVIIR  VRPTFQGNGLNGELIR  LLLEKNASVNEVDFEGR |
| Splice isoform 2 of signal transducer and activator of transcription 1-alpha/beta | IPI00218188 | 2 | VMNMEESTNGSLAAEFR  YYSRPKEAPEPMELDGPKGTGYIK |
| Splice isoform 2 of tripartite motif protein 7 | IPI00386829 | 3 | VWNVSLK  LGERAQDLPNHPCR  QNENLAQLGVEITQLSK |
| Splice isoform 2 of voltage-dependent N-type calcium channel alpha-1B subunit | IPI00220431 | 10 | VLRPLK  QGYTIR  HLRPDMK  GPGPEGGRRHHR  RFCHYIVTMR  AEAPKAESGEPGAR  TESSSYFRRKEK |
| Splice isoform 3 of amyloid beta A4 protein precursor | IPI00219183 | 4 | SQVMTHLR  EWEEAER  LALENYITALQAVPPRPR |
| Splice isoform 3 of dystrophin | IPI00220577 | 11 | QQHEHK  TGIISLCK  AKLESWK  IEHYASR  LLQVAVEDR  LGLLLHDSIQIPR  EGPYTVDAIQKKITETK |
| Splice isoform 3 of fibronectin precursor | IPI00339223 | 15 | ATITGYR  ITGYIIK  SDTVPSPR  SEPLIGRKK  GDSPASSKPISINYR  VTDATETTITISWR  YSFCTDHTVLVQTR  HTSVQTTSSGSGPFTDVR  LDAPTNLQFVNETDSTVLVR |
| Splice isoform 3 of kinesin-like motor protein KIF16B | IPI00452248 | 12 | FREMHK  LASLNSGSR  SDKAELER  LKEGGNINK  CEHDKESR  YVLCGQGKDAHFEFEVK  DILKKEVQEEQEILECLK |
| Splice isoform 3 of myosin Va | IPI00220154 | 3 | QGGSPVIEGVDDAKEMAHTR  AGQVAYLEKLRADKLRAACIR  SSALDYHELNEDGELWLVYEGLK |
| Splice isoform 3 of myosin VIIa | IPI00215754 | 5 | TTQIFHK  SYFPSIPK  GKDRLWSHTREPLK |
| Splice isoform 3 of neuronal cell adhesion molecule precursor | IPI00333778 | 15 | GHLQGYR  DYIIDPR  ILTFQGSK  QPEYAVVQR  LSPYVNYSFR  NALGAIHHTISVR  SLPSEASEQYLTK  VFNTPEGVPSAPSSLK  YIVSGTPTFVPYLIK |
| Splice isoform 3 of neurotrimin precursor | IPI00442298 | 2 | AVGFVSEDEYLEIQGITR  EQSGDYECSASNDVAAPVVR |
| Splice isoform 4 of calpain 10 | IPI00220234 | 7 | VGQTAGGSR  LHAADWAGR  NNSGFPSNPK  VEKRRVNLPR  QLLHLKDQCLISCCVLSPR |
| Splice isoform 4 of fibronectin precursor | IPI00339224 | 6 | VTPKEK  AQITGYR  STTPDITGYR  GDSPASSKPISINYR |
| Splice isoform 4 of Golgi autoantigen, golgin subfamily A member 4 | IPI00220522 | 5 | QEQEDLELK  LQHFQELGEEKDNR  SAKNVAAYTEQEEADSQGCVQK  VEMEELTSKYEKLQALQQMDGR  ILLFGCEKEEMNKEITWLKEEGVK |
| Splice isoform 4 of Neuronal cell adhesion molecule precursor | IPI00415032 | 5 | KEIVNGSR  QPEYAVVQR  ISWLTNGVPIEIAPDDPSR  VPLILFLCQMISALEVPLDPK |
| Splice isoform 5 of amyloid beta A4 protein precursor | IPI00219185 | 3 | GLTTRPGSGLTNIK  HVFNMLKKYVR  CLVGEFVSDALLVPDKCK |
| Splice isoform 7 of myelin-oligodendrocyte glycoprotein precursor | IPI00376382 | 2 | TELLKDAIGEGKVTLR  ISPGKNATGMEVGWYRPPFSR |
| Superoxide dismutase 1, soluble | IPI00218733 | 4 | LACGVIGIAQ  GDGPVQGIINFEQK |
| TAR RNA loop binding protein | IPI00298447 | 2 | LDQNFLNGIIDRIFQAGFTNNQASIK  ILPFSPEFSEFIIGPLMDALSESSLYSR |
| Taste receptor type 2 member 16 | IPI00027216 | 2 | LMPVDMILISLGISR  QIQHHSTGHCNPSMKAR |
| Taste receptor type 2 member 7 | IPI00028328 | 2 | LFLNLATLLPFCVCLMSFFLLILSLR  VISWILLGCVVLSVFISLPATENLNADFR |
| Thioesterase superfamily member 2 | IPI00020530 | 2 | NFERVLGK  TLAFTSVDLTNK |
| Titin | IPI00179357 | 2 | DELLHWTKELTEEEKKALAEEGK  VPEVPKKPEEKVPVLIPKKEKPPPAK |
| TNFSF10 protein | IPI00000049 | 6 | NEKALGRK  QQNISPLVR  SGHSFLSNLHLRNGELVIHEK |
| Transforming growth factor-beta induced protein IG-H3 precursor | IPI00018219 | 3 | EGVYTVFAPTNEAFR  YHIGDEILVSGGIGALVR  FSMLVAAIQSAGLTETLNR |
| Transthyretin precursor | IPI00022432 | 5 | VEIDTK  YTIAALLSPYSYSTTAVVTNPE  RYTIAALLSPYSYSTTAVVTNPK |
| TRIF-related adapter molecule | IPI00329281 | 2 | INSCPLSLSWGKR  GFPTQVERIFQESVYK |
| Trypsin I precursor | IPI00011694 | 2 | VSTISLPTAPPATGTK  LGEHNIEVLEGNEQFINAAK |
| Tu translation elongation factor, mitochondrial | IPI00027107 | 3 | SLERAEAGDNLGALVR  KYEEIDNAPEERAR  RGLVMVKPGSIKPHQK |
| Tumor necrosis factor receptor superfamily member 8 precursor | IPI00006073 | 4 | LAQEAASK  SGASVTEPVAEER  LHLCYPVQTSQPK  TCECRPGMICATSATNSCAR |
| Tumor necrosis factor, alpha-induced protein 3 | IPI00009448 | 4 | RQKNQNKLPK  TTQSTSRPKCAR  LNSKPGPEGLPGMALGASR |
| Tyrosine phosphatase zeta polypeptide 2 HTPZP2 | IPI00472466 | 2 | KAAYAKRHAVGPVVVHCSAGVGR SDAGLVGGGEDGDTDDDGDDDDDDR |
| Ubiquitin carboxyl-terminal hydrolase isozyme L1 | IPI00018352 | 7 | VYFMK  QFLSETEK  GQEVSPK  EFTEREQGEVR |
| Vitamin D-binding protein precursor | IPI00298853 | 6 | LSNLIK  EFSHLGK  ELSSFIDK  LAQKVPTADLEDVLPLAEDITNILSK |
| Vitamin K-dependent protein S precursor | IPI00294004 | 6 | VYFAGFPR  FSAEFDFR  QSTNAYPDLR  SFQTGLFTAAR  SQDILLSVENTVIYR |
| Vitronectin precursor | IPI00298971 | 7 | VYFFK  AVRPGYPK  FEDGVLDPDYPR  DVWGIEGPIDAAFTR  SIAQYWLGCPAPGHL |
| WD repeat and FYVE domain containing protein 1 | IPI00024283 | 8 | SGNMLGR  QHHCRK  TYPAHQNR  VCDSCYDSIK  LEQNTCSVITTLKc |
| XPR1 protein | IPI00217110 | 2 | GGFLLIEFLFLLGINTYGWR  LAFSEFYLSLILLQNYQNLNFTGFRK |
| Zinc finger MYND domain containing protein 19 | IPI00061171 | 2 | IFAYAFDKNR  LDNLQLVPWGWRPKAEETSSK |
| Ceruloplasmin precursor | IPI00017601 | 10 | IGGSYK  TYSDHPEK  EYTDASFTNR  DLYSGLIGPLIVCR  DIASGLIGPLIICK  VNKDDEEFIESNK  GVYSSDVFDIFPGTYQTLEMFPR |
| Epsilon globin | IPI00217471 | 2 | LLVVYPWTQR  EFTPEVQAAWQK |
| Follistatin-like 4 | IPI00477747 | 4 | LLVESLFR  YIYVAQPALSR  GPDVGVGESQAEEPR |
| Hypothetical protein LOC122618 | IPI00060310 | 6 | FWVVDGR  MPPRRPWDR  TSTDLQVLAAR  YWPVLDNALRAAAFGKGVR |
| LOC400684 protein | IPI00452693 | 2 | AAVAGSGVR  TQSPATSGR |
| Neuronal pentraxin receptor isoform 1 | IPI00334238 | 3 | ELDVLQGR  MDQLEGQLLAQVLALEK |
| Nuclear pore complex protein Nup93 | IPI00397904 | 7 | AVYCIIGR  ESMLVEWEQVK  CHAVHVALVLFELK  TWFQEYMNSKDRR  EALQYFYFLRDEKDSQGENMFLR |
| Splice isoform 3 of seizure 6-like protein precursor | IPI00220333 |  | GVDGPTLTVLANQTLLVEGQVIR  SALLYDSLQTESVPFEGLLSEGNTIR  VLSPSYPENTNGSQFCIWTIEAPEGQK |

**^#^** International Protein Index (www.ebi.ac.uk/IPI)

* the number of identified peptides
