# Supplementary material for: Identification of the soluble form of tyrosine kinase receptor Axl as a potential biomarker for intracranial aneurysm rupture
Source: BMC Neurol. 2015 Mar 5;15:23. doi: 10.1186/s12883-015-0282-8 (PMC4375882; doi:10.1186/s12883-015-0282-8)
Supplement: Additional file 3: — Proteins identified in human CSF. [file 12883_2015_282_MOESM3_ESM.doc]

**Additional file 3. Proteins identified in human CSF**

| **Protein Name** | **IPI #** | **Uniprot #** |
| --- | --- | --- |
| 17-beta hydroxysteroid dehydrogenase | IPI00376206 | Q7Z5P4 |
| 24 kDa protein | IPI00479531 | P02763 |
| 36 kDa protein | IPI00477751 | P43234 |
| 376 kDa protein | IPI00479143 | O95613-1 |
| 39 kDa protein | IPI00479497 | Q7Z3B1 |
| 56 kDa protein | IPI00479747 | Q96KN2 |
| 66 kDa protein | IPI00472812 | Q6ZT98-1 |
| Acheron, isoform 1 | IPI00414661 | Q9BRS8 |
| Adrenomedullin 2 precursor | IPI00385109 | Q7Z4H4 |
| Afamin precursor | IPI00019943 | P43652 |
| Alcadein alpha-1 | IPI00007257 | O94985-2 |
| Alpha-1-acid glycoprotein 2 precursor | IPI00020091 | P19652 |
| Alpha-1-antitrypsin precursor | IPI00305457 | Q9P173 |
| Alpha-1B-glycoprotein precursor | IPI00022895 | P04217-1 |
| Alpha-2-glycoprotein 1, zinc | IPI00166729 | P25311 |
| Alpha-2-HS-glycoprotein precursor | IPI00022431 | C9JV77 |
| Alpha-2-macroglobulin precursor | IPI00478003 | [P01023](http://srs.ebi.ac.uk/srsbin/cgi-bin/wgetz?-id+4xQIW1hZBxx+%5Bswissprot-AccNumber:P01023%5D+-e) |
| AMBP protein precursor | IPI00022426 | P02760 |
| Amyloid beta A4 precursor protein-binding family A member 1 | IPI00294556 | Q02410 |
| Amyloid-like protein 1 precursor | IPI00020012 | P51693-1 |
| Angiotensinogen precursor | IPI00032220 | P01019 |
| Ankyrin repeat and FYVE domain protein 1 | IPI00159899 | Q9P2R3 |
| Antithrombin III variant | IPI00032179 | P01008 |
| Apolipoprotein A-I precursor | IPI00021841 | P02647 |
| Apolipoprotein A-II precursor | IPI00021854 | P02652 |
| Apolipoprotein A-IV precursor | IPI00304273 | P06727 |
| Apolipoprotein D precursor | IPI00006662 | P05090 |
| Apolipoprotein E precursor | IPI00021842 | P02649 |
| ASRGL1 protein | IPI00169322 | Q7L266-1 |
| AXL receptor tyrosine kinase, isoform 1 | IPI00296992 | P30530-1 |
| Baculoviral IAP repeat-containing protein 4 | IPI00303890 | P98170 |
| BAG-family molecular chaperone regulator-3 | IPI00000644 | O95817 |
| Beta-2-glycoprotein I precursor | IPI00298828 | P02749 |
| Beta-2-microglobulin precursor | IPI00004656 | P61769 |
| Biotinidase precursor | IPI00218413 | P43251 |
| C4B1 | IPI00418163 | B4E344 |
| Calreticulin precursor | IPI00020599 | P27797 |
| Calsyntenin-1 precursor | IPI00413959 | O94985-1 |
| Carboxypeptidase E precursor | IPI00031121 | P16870-1 |
| Cathepsin D precursor | IPI00011229 | P07339 |
| Cathepsin L precursor | IPI00012887 | P07711 |
| CD59 glycoprotein precursor | IPI00011302 | [P13987](http://srs.ebi.ac.uk/srsbin/cgi-bin/wgetz?-id+4xQIW1hZBXi+%5Bswissprot-AccNumber:P13987%5D+-e) |
| Cell surface glycoprotein MUC18 precursor | IPI00016334 | [P43121-1](http://srs.ebi.ac.uk/srsbin/cgi-bin/wgetz?-id+4xQIW1hZBWi+%5Bswissprot-AccNumber:P43121%5D+-e) |
| Chitinase-3 like protein 1 precursor | IPI00002147 | P36222 |
| Chromosome 1 open reading frame 27 | IPI00305788 | Q5SWX8-1 |
| Clusterin isoform 1 | IPI00400826 | P10909-2 |
| Clusterin precursor | IPI00291262 | P10909-1 |
| Coagulation factor XII precursor | IPI00019581 | P00748 |
| Collagen alpha 1(I) chain precursor | IPI00297646 | P02452 |
| Collagen alpha 1(VI) chain precursor | IPI00291136 | P12109 |
| Collagen alpha 2(I) chain precursor | IPI00304962 | P08123 |
| Complement C1q subcomponent, C chain precursor | IPI00022394 | P02747 |
| Complement C1s subcomponent precursor | IPI00017696 | P09871 |
| Complement C3 precursor | IPI00164623 | P01024 |
| Complement C5 precursor | IPI00032291 | [P01031](http://srs.ebi.ac.uk/srsbin/cgi-bin/wgetz?-id+4xQIW1hZBIh+%5Bswissprot-AccNumber:P01031%5D+-e) |
| Complement component C6 precursor | IPI00009920 | P13671 |
| Complement component C7 precursor | IPI00296608 | P10643 |
| Complement component C9 precursor | IPI00022395 | P02748 |
| Complement factor I precursor | IPI00291867 | P05156 |
| Condensin subunit 2 | IPI00299507 | Q15003 |
| Contactin 2 precursor | IPI00024966 | Q02246 |
| Cortactin-binding protein 2 | IPI00103869 | Q8WZ74 |
| Corticosteroid-binding globulin precursor | IPI00027482 | P08185 |
| Cystatin C precursor | IPI00032293 | P01034 |
| Death-associated protein kinase 1 | IPI00021250 | B4DHI4 |
| Dickkopf related protein-3 precursor | IPI00002714 | Q9UBP4 |
| DJ788L20.2 | IPI00100250 | Q9HCC6 |
| DOC-2/DAB2 interactive protein | IPI00395467 | Q5VWQ8-4 |
| Dolichyl-P-Man:Man | IPI00012208 | Q9BV10 |
| Ephrin A1 isoform b prEcursor | IPI00377015 | P20827-2 |
| Ephrin-B2 precursor | IPI00005126 | P52799 |
| Epidermal growth factor receptor substrate 15 | IPI00292134 | P42566 |
| Extracellular matrix protein 1 | IPI00006969 | Q16610-2 |
| Extracellular superoxide dismutase [Cu-Zn] precursor | IPI00027827 | P08294 |
| FLJ00385 protein | IPI00168728 | Q8NF17 |
| Full-length cDNA clone CS0DN001YP04 of Adult brain of Homo sapiens | IPI00383975 | Q86T07 |
| Galectin-3 binding protein precursor | IPI00023673 | Q08380 |
| Gamma-taxilin | IPI00019994 | Q9NUQ3-1 |
| Gelsolin precursor | IPI00026314 | P06396 -1 |
| Glucocorticoid receptor DNA bindinG factor 1 isoform b | IPI00477498 | Q9NRY4-1 |
| Glutamate decarboxylase, 65 kDa isoform | IPI00012796 | Q05329 |
| Grb10 interacting GYF protein 1 | IPI00428657 | O75420 |
| Guanylate binding protein 4 | IPI00419541 | Q96PP9 |
| H2AFY protein | IPI00304171 | O75367-1 |
| Haptoglobin precursor | IPI00478493 | Q0VAC5 |
| Hemoglobin gamma-G | IPI00464992 | P69892 |
| Hemopexin precursor | IPI00022488 | P02790 |
| Hepatoma-derived growth factor-related protein 2, isoform 2 | IPI00013290 | Q7Z4V5-2 |
| HMG-box containing protein | IPI00300222 | Q8WY36-1 |
| Hypothetical protein | IPI00026195 | P06310 |
| Hypothetical protein | IPI00384355 | P0CG05 |
| Hypothetical protein | IPI00395435 | Q5NV90 |
| Hypothetical protein | IPI00430804 | Q6GMV7 |
| Hypothetical protein DKFZp313D1622 | IPI00296554 | Q5VZM2-2 |
| Hypothetical protein DKFZp434C011 | IPI00152946 | Q9H0H5 |
| Hypothetical protein DKFZp434O194 | IPI00031282 | Q9H0B3-1 |
| Hypothetical protein DKFZp686A05192 | IPI00375705 | Q5TFQ8 |
| Hypothetical protein DKFZp686C15213 | IPI00426051 | Q6MZU6 |
| Hypothetical protein DKFZp686D0623 | IPI00470584 | Q8NEK8 |
| Hypothetical protein DKFZp686G11190 | IPI00426007 | Q6MZQ6 |
| Hypothetical protein DKFZp686G21220 | IPI00423460 | Q6N090 |
| Hypothetical protein DKFZp686I04196 | IPI00399007 | [P01859](http://srs.ebi.ac.uk/srsbin/cgi-bin/wgetz?-id+4xQIW1hZ86g+%5Bswissprot-AccNumber:P01859%5D+-e) |
| Hypothetical protein DKFZp686I15212 | IPI00418153 | Q6N030 |
| Hypothetical protein DKFZp761H2017 | IPI00166552 | Q8N3G9-2 |
| Hypothetical protein FLJ11594 | IPI00386962 | Q8N3J3-2 |
| Hypothetical protein FLJ11633 | IPI00386957 | Q9H9A5-3 |
| Hypothetical protein FLJ20475 | IPI00183065 | Q9NX28 |
| Hypothetical protein FLJ22037 | IPI00025094 | Q9H6N6 |
| Hypothetical protein FLJ23322 | IPI00166509 | Q8IWF2-1 |
| Hypothetical protein FLJ23757 | IPI00154836 | Q8TE96-1 |
| Hypothetical protein FLJ25298 | IPI00386524 | Q96DK0 |
| Hypothetical protein FLJ34458 | IPI00300753 | Q496Y0-1 |
| Hypothetical protein FLJ35721 | IPI00167940 | Q8NA97 |
| Hypothetical protein FLJ41598 | IPI00419164 | A8K2U0 |
| Hypothetical protein FLJ42206 | IPI00446339 | Q14BN4-6 |
| Hypothetical protein FLJ45525 | IPI00299571 | Q15084-2 |
| Hypothetical protein FLJ46072 | IPI00394829 | Q6ZRV2 |
| Hypothetical protein FLJ90661 | IPI00168352 | Q5K4E3 |
| Hypothetical protein KIAA0156 | IPI00006025 | Q15020-1 |
| Hypothetical protein KIAA0792 | IPI00477163 | O94886 |
| Hypothetical protein MGC10992 | IPI00059169 | Q96A19 |
| Hypothetical protein pp6318 | IPI00103828 | B1AJZ5 |
| Ig kappa chain V-III region WOL | IPI00387118 | P01623 |
| Ig kappa chain V-IV region B17 precursor | IPI00386133 | P06314 |
| Immunoglobulin-like domain protein MGC33530 precursor | IPI00290411 | Q8TAG5-2 |
| Importin 9 | IPI00185146 | Q96P70 |
| Insulin-like growth factor binding protein 7 precursor | IPI00016915 | Q16270 |
| Inter-alpha-trypsin inhibitor heavy chain H1 precursor | IPI00292530 | P19827 |
| Inter-alpha-trypsin inhibitor heavy chain H2 precursor | IPI00305461 | P19823 |
| Isg20 protein | IPI00031824 | Q59F22 |
| ISLR precursor | IPI00023648 | O14498 |
| JRK protein | IPI00216621 | O75566 |
| Keratin 1 | IPI00220327 | P04264 |
| Keratin 9 | IPI00019359 | P35527 |
| KIAA0319 protein | IPI00006524 | Q5VV43-1 |
| KIAA0690 | IPI00101186 | Q5JTH9-1 |
| Laminin alpha-1 chain precursor | IPI00375294 | P25391 |
| Leucine-rich alpha-2-glycoprotein precursor | IPI00022417 | P02750 |
| Limbic system-associated membrane protein precursor | IPI00013303 | Q13449 |
| LP2209 | IPI00428724 | Q6XYC0 |
| Lumican precursor | IPI00020986 | P51884 |
| Lysosomal-associated membrane protein 2C | IPI00216172 | P13473 |
| Lysosomal-associated multitransmembrane protein | IPI00013827 | Q13571 |
| Metalloproteinase inhibitor 1 precursor | IPI00032292 | P01033 |
| Microtubule associated serine/threonine kinase-like | IPI00074258 | Q96GX5-1 |
| Mimecan precursor | IPI00025465 | P20774 |
| Monocyte differentiation antigen CD14 precursor | IPI00029260 | P08571 |
| MUF1 protein | IPI00397576 | Q9H822 |
| Mutated in bladder cancer 1 | IPI00168663 | Q8NEF3-1 |
| Myosin heavy chain, cardiac muscle alpha isoform | IPI00302328 | P13533 |
| N-acetylgalactosamine-4-O-sulfotransferase | IPI00300838 | Q9H2A9 |
| N-acetyllactosaminide beta-1,3-N-acetylglucosaminyltransferase | IPI00009997 | O43505 |
| N-acetyltransferase 5 isoform c | IPI00375483 | A6NHA3 |
| Nebulin | IPI00303335 | P20929 |
| Nectin-like protein 2 | IPI00003813 | Q9BY67-1 |
| Neural cell adhesion molecule | IPI00299059 | O00533-2 |
| Neural cell adhesion molecule 1, 140 kDa isoform precursor | IPI00435020 | P13591-1 |
| Neural cell adhesion molecule 2 | IPI00478109 | O15394 |
| Neuroblastoma suppressor of tumorigenicity 1 precursor | IPI00013299 | [P41271-1](http://srs.ebi.ac.uk/srsbin/cgi-bin/wgetz?-id+4xQIW1hZ72U+%5Bswissprot-AccNumber:P41271%5D+-e) |
| Neurocan core protein precursor | IPI00159927 | O14594 |
| NeuroNal peNtraxiN I precursor | IPI00220562 | Q15818 |
| Neurosecretory protein VGF precursor | IPI00289501 | O15240 |
| NICE-4 protein | IPI00005416 | Q9UGL4 |
| NifU-like protein HIRIP5 | IPI00455153 | Q9UMS0-1 |
| Nogo receptor-like 3 | IPI00328746 | Q86UN3 |
| Nucleolysin TIAR | IPI00005615 | Q01085 |
| Obscurin | IPI00479915 | Q5VST9-5 |
| Opioid binding protein/cell adhesion molecule precursor | IPI00001662 | B7ZLQ1 |
| OTTHUMP00000022089 | IPI00170641 | Q8TEH3-1 |
| P protein | IPI00028627 | Q04671-1 |
| Peroxisomal targeting signal 1 receptor | IPI00032931 | P50542-2 |
| Phosphatidylcholine-sterol acyltransferase precursor | IPI00022331 | P04180 |
| Pigment epithelium-derived factor precursor | IPI00006114 | P36955 |
| Plasma kallikrein precursor | IPI00008558 | P03952 |
| Plasma protease C1 inhibitor precursor | IPI00291866 | P05155 |
| Plasminogen precursor | IPI00019580 | P00747 |
| PREDICTED: chromosome 20 open reading frame 142 | IPI00374076 | Q8N6M3 |
| PREDICTED: dynein, cytoplasmic, heavy polypeptide 2 | IPI00171494 | Q8NCM8-2 |
| PREDICTED: hypothetical protein XP_291007 | IPI00216817 | A6NES4 |
| PREDICTED: KIAA0146 protein | IPI00029021 | Q14159 |
| PREDICTED: KIAA1509 | IPI00029170 | Q9P219-2 |
| PREDICTED: KIAA1522 protein | IPI00001632 | Q9P206-2 |
| PREDICTED: odz, odd Oz/ten-m homolog 3 | IPI00398020 | Q9P273 |
| PREDICTED: similar to RIKEN cDNA 1700022C21 | IPI00400925 | Q5SQS8 |
| PREDICTED: similar to TAR DNA binding protein | IPI00147770 | B1AKP7 |
| PREDICTED: similar to tumor necrosis factor, alpha-induced protein 2 | IPI00073442 | Q17RC7 |
| PRF1 protein | IPI00293423 | P14222 |
| Prion protein | IPI00382843 | Q6SES1 |
| Procollagen C-proteinase enhancer protein precursor | IPI00299738 | Q15113 |
| Progesterone-induced blocking factor 1 | IPI00472584 | Q8WXW3-1 |
| Prostaglandin-H2 D-isomerase precursor | IPI00013179 | P41222 |
| Protein F25965 | IPI00062869 | Q96GY3 |
| Protein kinase C-binding protein NELL2 precursor | IPI00015260 | Q99435 |
| Protein tyrosine PhosPhatase, non-recePtor tyPe substrate 1 Precursor | IPI00332887 | P78324-1 |
| Prothrombin precursor | IPI00019568 | P00734 |
| PTPL1-associated RhoGAP | IPI00152011 | Q52LW3-1 |
| Receptor-interacting serine/threonine-protein kinase 2 | IPI00021917 | O43353-1 |
| Retinoblastoma-associated factor 600 | IPI00180305 | Q5T4S7-5 |
| RGD, leucine-rich repeat, tropomodulin and proline-rich containing protein | IPI00456628 | Q6F5E8 |
| Ribonuclease pancreatic precursor | IPI00014048 | P07998 |
| SEC14 and spectrin domains 1 | IPI00329002 | Q86VW0 |
| Secretogranin I precursor | IPI00006601 | P05060 |
| Secretogranin II precursor | IPI00009362 | P13521 |
| Secretogranin III precursor | IPI00292071 | Q8WXD2 |
| Selenoprotein S | IPI00020468 | Q9BQE4 |
| Semaphorin 7A precursor | IPI00025257 | O75326 |
| Septin 10 iSoform 2 | IPI00412153 | F5H1F2 |
| Serine (or cySteine) proteinaSe inhibitor, clade A (alpha-1 antiproteinaSe, antitrypSin | IPI00396348 | P29622 |
| Serotransferrin precursor | IPI00022463 | P02787 |
| SERPINC1 protein | IPI00165421 | P01008 |
| SERPIND1 protein | IPI00292950 | P05546 |
| Serum albumin precursor | IPI00022434 | A8K9P0 |
| SEZ6L2 protein | IPI00306470 | Q8NC33 |
| Similar to peptide N-glycanase homolog | IPI00165496 | Q96IV0-2 |
| Small intestine SPAK-like kinase | IPI00457335 | Q9UEW8 |
| SPARC-like protein 1 precursor | IPI00296777 | Q14515 |
| Spectrin beta chain, brain 4 | IPI00219168 | Q9NRC6 |
| Splice Isoform 1 Of Adenomatous polyposis coli protein | IPI00012391 | P25054-1 |
| Splice Isoform 1 Of Amine oxidase flavin containing domain protein 2 | IPI00456631 | O60341-1 |
| Splice Isoform 1 Of B-lymphocyte antigen precursor | IPI00024024 | Q02040-1 |
| Splice Isoform 1 Of Brevican core protein precursor | IPI00456623 | Q96GW7-1 |
| Splice Isoform 1 Of Complement factor B precursor | IPI00019591 | B4E1Z4 |
| Splice Isoform 1 Of Complement factor H precursor | IPI00029739 | P08603-1 |
| Splice Isoform 1 Of Contactin 1 precursor | IPI00029751 | Q12860-1 |
| Splice Isoform 1 Of COP9 signalosome complex subunit 1 | IPI00479323 | Q13098-7 |
| Splice Isoform 1 Of Ecto-ADP-ribosyltransferase 3 precursor | IPI00013682 | Q13508-1 |
| Splice Isoform 1 Of EGF-containing fibulin-like extracellular matrix protein 1 precurso | IPI00029658 | Q12805-1 |
| Splice Isoform 1 Of Erythrocyte membrane protein band 4.2 | IPI00028614 | P16452-1 |
| Splice Isoform 1 Of Fibrinogen gamma chain precursor | IPI00021891 | P02679-1 |
| Splice Isoform 1 Of Fibulin-1 precursor | IPI00296534 | P23142-1 |
| Splice Isoform 1 Of High-affinity cGMP-specific 3',5'-cyclic phosphodiesterase 9A | IPI00008806 | O76083-1 |
| Splice Isoform 1 Of Inter-alpha-trypsin inhibitor heavy chain H4 precursor | IPI00294193 | Q14624-1 |
| Splice Isoform 1 Of Neogenin precursor | IPI00023814 | Q92859-1 |
| Splice Isoform 1 Of Proactivator polypeptide precursor | IPI00012503 | P07602-1 |
| Splice Isoform 1 Of Serologically defined colon cancer antigen 1 | IPI00301618 | O60524-1 |
| Splice Isoform 1 Of Tetratricopeptide repeat protein 7A | IPI00397195 | Q9ULT0-1 |
| Splice Isoform 1 Of Ubiquitin carboxyl-terminal hydrolase 6 | IPI00423562 | P35125-1 |
| Splice Isoform 2 Of Adenosine kinase | IPI00234368 | P55263-2 |
| Splice Isoform 2 Of Contactin 1 precursor | IPI00216641 | Q12860-2 |
| Splice Isoform 2 Of Ectonucleotide pyrophosphatase/phosphodiesterase 2 | IPI00303210 | Q13822-2 |
| Splice Isoform 2 Of Fibrinogen alpha/alpha-E chain precursor | IPI00029717 | P02671-2 |
| Splice Isoform 2 Of Interleukin-17E precursor | IPI00332192 | Q9H293-2 |
| Splice Isoform 2 Of Kininogen precursor | IPI00215894 | P01042-2 |
| Splice Isoform 2 Of Neuronal-specific septin 3 | IPI00384187 | Q9UH03-2 |
| Splice Isoform 2 Of Phospholipid transfer protein precursor | IPI00217778 | P55058-2 |
| Splice Isoform 2 Of Poliovirus receptor related protein 1 precursor | IPI00218887 | Q15223-2 |
| Splice Isoform 2 Of Serine/threonine-protein kinase RIPK4 | IPI00215935 | P57078-2 |
| Splice Isoform 2 Of Signal transducer and activator of transcription 1-alpha/beta | IPI00218188 | P42224-2 |
| Splice Isoform 2 Of Tripartite motif protein 7 | IPI00386829 | Q9C029-2 |
| Splice Isoform 2 Of Voltage-dependent N-type calcium channel alpha-1B subunit | IPI00220431 | Q00975-2 |
| Splice Isoform 3 Of Amyloid beta A4 protein precursor | IPI00219183 | P05067-3 |
| Splice Isoform 3 Of Dystrophin | IPI00220577 | P11532-3 |
| Splice Isoform 3 Of Fibronectin precursor | IPI00339223 | P02751-3 |
| Splice Isoform 3 Of Kinesin-like motor protein KIF16B | IPI00452248 | Q96L93-3 |
| Splice Isoform 3 Of Myosin Va | IPI00220154 | Q9Y4I1-3 |
| Splice Isoform 3 Of Myosin VIIa | IPI00215754 | Q13402-3 |
| Splice Isoform 3 Of Neuronal cell adhesion molecule precursor | IPI00333778 | Q92823-3 |
| Splice Isoform 3 Of Neurotrimin precursor | IPI00442298 | Q9P121-3 |
| Splice Isoform 4 Of Calpain 10 | IPI00220234 | Q9HC96-4 |
| Splice Isoform 4 Of Fibronectin precursor | IPI00339224 | P02751-4 |
| Splice Isoform 4 Of Golgi autoantigen, golgin subfamily A member 4 | IPI00220522 | Q13439-4 |
| Splice Isoform 4 Of Neuronal cell adhesion molecule precursor | IPI00415032 | Q92823-4 |
| Splice Isoform 5 Of Amyloid beta A4 protein precursor | IPI00219185 | P05067-5 |
| Splice Isoform 7 Of Myelin-oligodendrocyte glycoprotein precursor | IPI00376382 | Q16653-7 |
| Superoxide diSmutaSe 1, Soluble | IPI00218733 | P00441 |
| TAR RNA loop binding protein | IPI00298447 | Q13395 |
| Taste receptor type 2 member 16 | IPI00027216 | Q9NYV7 |
| Taste receptor type 2 member 7 | IPI00028328 | Q9NYW3 |
| Thioesterase superfamily member 2 | IPI00020530 | Q9NPJ3 |
| Titin | IPI00179357 | Q8WZ42 |
| TNFSF10 protein | IPI00000049 | P50591 |
| Transforming growth factor-beta induced protein IG-H3 precursor | IPI00018219 | Q15582 |
| Transthyretin precursor | IPI00022432 | P02766 |
| TRIF-related adapter molecule | IPI00329281 | Q86XR7 |
| Trypsin I precursor | IPI00011694 | P07477 |
| Tu translation elongation factor, mitochondrial | IPI00027107 | P49411 |
| Tumor necrosis factor receptor superfamily member 8 precursor | IPI00006073 | P28908 |
| Tumor necrosis factor, alpha-induced protein 3 | IPI00009448 | P21580 |
| Tyrosine phosphatase zeta polypeptide 2 HTPZP2 | IPI00472466 | P23471 |
| Ubiquitin carboxyl-terminal hydrolase isozyme L1 | IPI00018352 | P09936 |
| Vitamin D-binding protein precursor | IPI00298853 | P02774 |
| Vitamin K-dependent protein S precursor | IPI00294004 | P07225 |
| Vitronectin precursor | IPI00298971 | P04004 |
| WD repeat and FYVE domain containing protein 1 | IPI00024283 | Q8IWB7 |
| XPR1 protein | IPI00217110 | Q9UBH6 |
| Zinc finger MYND domain containing protein 19 | IPI00061171 | Q96E35 |
| Ceruloplasmin precursor | IPI00017601 | P00450 |
| Epsilon globin | IPI00217471 | P02100 |
| Follistatin-like 4 | IPI00477747 | Q6MZW2 |
| Hypothetical protein LOC122618 | IPI00060310 | B4DI07 |
| LOC400684 protein | IPI00452693 | Q9BVU7 |
| NeuroNal peNtraxiN receptor isoform 1 | IPI00334238 | O95502 |
| Nuclear pore complex protein Nup93 | IPI00397904 | Q8N1F7 |
| Splice Isoform 3 Of Seizure 6-like protein precursor | IPI00220333 | Q9BYH1 |

**IPI**: International Protein Index (www.ebi.ac.uk/IPI)

**Uniprot**: Protein knowledgebase (www.uniprot.org)
