# Supplementary material for: Identification of the soluble form of tyrosine kinase receptor Axl as a potential biomarker for intracranial aneurysm rupture
Source: BMC Neurol. 2015 Mar 5;15:23. doi: 10.1186/s12883-015-0282-8 (PMC4375882; doi:10.1186/s12883-015-0282-8)
Supplement: Additional file 4: — Proteins annotated as glycoproteins in UniprotKB. [file 12883_2015_282_MOESM4_ESM.doc]

**Additional file 4.**  Proteins annotated as glycoproteins in UniprotKB

| **Protein Name** | **Uniprot** | **glycosylation** | **Secreted** |
| --- | --- | --- | --- |
| 24 kDa protein | P02763 | N-linked | Yes |
| 36 kDa protein | P43234 | N-linked | ? |
| 56 kDa protein | Q96KN2 | N-linked | Yes |
| Afamin precursor | P43652 | N-linked | yes |
| Alcadein alpha-1 | O94985-2 | N-linked | yes |
| Alpha-1-acid glycoprotein 2 precursor | P19652 | N-linked | yes |
| Alpha-1B-glycoprotein precursor | P04217-1 | N-linked | yes |
| Alpha-2-glycoprotein 1, zinc | P25311 | N-linked | yes |
| Alpha-2-macroglobulin precursor | [P01023](http://srs.ebi.ac.uk/srsbin/cgi-bin/wgetz?-id+4xQIW1hZBxx+%5Bswissprot-AccNumber:P01023%5D+-e) | N-linked | yes |
| Angiotensinogen precursor | P01019 | N-linked | yes |
| Antithrombin III variant | P01008 | N-linked | yes |
| Apolipoprotein A-I precursor | P02647 | N-linked | yes |
| Apolipoprotein D precursor | P05090 | N-linked | yes |
| AXL receptor tyrosine kinase, isoform 1 | P30530-1 | N-linked | yes |
| Beta-2-microglobulin precursor | P61769 | N-linked | yes |
| Biotinidase precursor | P43251 | N-linked | yes |
| Calreticulin precursor | P27797 | N-linked | yes |
| Calsyntenin-1 precursor | O94985-1 | N-linked | yes |
| Carboxypeptidase E precursor | P16870-1 | N-linked | yes |
| Cathepsin D precursor | P07339 | N-linked | yes |
| Cathepsin L precursor | P07711 | N-linked | yes |
| Cell surface glycoprotein MUC18 precursor | [P43121-1](http://srs.ebi.ac.uk/srsbin/cgi-bin/wgetz?-id+4xQIW1hZBWi+%5Bswissprot-AccNumber:P43121%5D+-e) | N-linked | yes |
| Chitinase-3 like protein 1 precursor | P36222 | N-linked | yes |
| Clusterin isoform 1 | P10909-2 | N-linked | yes |
| Clusterin precursor | P10909-1 | N-linked | yes |
| Collagen alpha 1(VI) chain precursor | P12109 | N-linked | yes |
| Collagen alpha 2(I) chain precursor | P08123 | N-linked | yes |
| Complement C1s subcomponent precursor | P09871 | N-linked | yes |
| Complement C3 precursor | P01024 | N-linked | yes |
| Complement C5 precursor | [P01031](http://srs.ebi.ac.uk/srsbin/cgi-bin/wgetz?-id+4xQIW1hZBIh+%5Bswissprot-AccNumber:P01031%5D+-e) | N-linked | yes |
| Complement component C9 precursor | P02748 | N-linked | yes |
| Complement factor I precursor | P05156 | N-linked | yes |
| Corticosteroid-binding globulin precursor | P08185 | N-linked | yes |
| Dickkopf related protein-3 precursor | Q9UBP4 | N-linked | yes |
| Ephrin-B2 precursor | P52799 | N-linked | yes |
| Extracellular matrix protein 1 | Q16610-2 | N-linked | yes |
| Extracellular superoxide dismutase [Cu-Zn] precursor | P08294 | N-linked | yes |
| Galectin-3 binding protein precursor | Q08380 | N-linked | yes |
| Hypothetical protein DKFZp686A05192 | Q5TFQ8 | N-linked | yes |
| Hypothetical protein DKFZp686I04196 | [P01859](http://srs.ebi.ac.uk/srsbin/cgi-bin/wgetz?-id+4xQIW1hZ86g+%5Bswissprot-AccNumber:P01859%5D+-e) | N-linked | yes |
| Hypothetical protein DKFZp761H2017 | Q8N3G9-2 | N-linked | yes |
| Hypothetical protein FLJ23322 | Q8IWF2-1 | N-linked | ? |
| Hypothetical protein FLJ41598 | A8K2U0 | N-linked | yes |
| Hypothetical protein FLJ90661 | Q5K4E3 | N-linked | yes |
| Hypothetical protein KIAA0792 | O94886 | N-linked | yes |
| Immunoglobulin-like domain protein MGC33530 precursor | Q8TAG5-2 | N-linked | yes |
| Insulin-like growth factor binding protein 7 precursor | Q16270 | N-linked | yes |
| ISLR precursor | O14498 | N-linked | yes |
| KIAA0319 protein | Q5VV43-1 | N-linked | yes |
| Laminin alpha-1 chain precursor | P25391 | N-linked | yes |
| Lumican precursor | P51884 | N-linked | yes |
| Metalloproteinase inhibitor 1 precursor | P01033 | N-linked | yes |
| N-acetylgalactosamine-4-O-sulfotransferase | Q9H2A9 | N-linked | yes |
| N-acetyllactosaminide beta-1,3-N-acetylglucosaminyltransferase | O43505 | N-linked | ? |
| Nectin-like protein 2 | Q9BY67-1 | N-linked | yes |
| Neural cell adhesion molecule | O00533-2 | N-linked | yes |
| Neural cell adhesion molecule 1, 140 kDa isoform precursor | P13591-1 | N-linked | yes |
| Neural cell adhesion molecule 2 | O15394 | N-linked | yes |
| Neurocan core protein precursor | O14594 | N-linked | yes |
| NeuroNal peNtraxiN I precursor | Q15818 | N-linked | ? |
| P protein | Q04671-1 | N-linked | ? |
| Plasma kallikrein precursor | P03952 | N-linked | yes |
| PREDICTED: odd Oz/ten-m homolog 3 | Q9P273 | N-linked | yes |
| PRF1 protein | P14222 | N-linked | yes |
| Procollagen C-proteinase enhancer protein precursor | Q15113 | N-linked | yes |
| Prostaglandin-H2 D-isomerase precursor | P41222 | N-linked | yes |
| Protein kinase C-binding protein NELL2 precursor | Q99435 | N-linked | yes |
| Protein tyrosine PhosPhatase, non-receptor tyPe substrate 1 Precursor | P78324-1 | N-linked | yes |
| Prothrombin precursor | P00734 | N-linked | yes |
| Ribonuclease pancreatic precursor | P07998 | N-linked | yes |
| Serine (or cySteine) proteinaSe inhibitor, clade A (alpha-1 antiproteinaSe, antitrypSin | P29622 | N-linked | yes |
| SERPINC1 protein | P01008 | N-linked | yes |
| SERPIND1 protein | P05546 | N-linked | yes |
| Splice Isoform 1 Of Complement factor H precursor | P08603-1 | N-linked | yes |
| Splice Isoform 1 Of Contactin 1 precursor | Q12860-1 | N-linked | yes |
| Splice Isoform 1 Of EGF-containing fibulin-like extracellular matrix protein 1 precurso | Q12805-1 | N-linked | yes |
| Splice Isoform 1 Of Fibrinogen gamma chain precursor | P02679-1 | N-linked | yes |
| Splice Isoform 1 Of Fibulin-1 precursor | P23142-1 | N-linked | yes |
| Splice Isoform 1 Of Neogenin precursor | Q92859-1 | N-linked | yes |
| Splice Isoform 1 Of Proactivator polypeptide precursor | P07602-1 | N-linked | yes |
| Splice Isoform 2 Of Contactin 1 precursor | Q12860-2 | N-linked | yes |
| Splice Isoform 2 Of Ectonucleotide pyrophosphatase/phosphodiesterase 2 | Q13822-2 | N-linked | yes |
| Splice Isoform 2 Of Fibrinogen alpha/alpha-E chain precursor | P02671-2 | N-linked | yes |
| Splice Isoform 2 Of Interleukin-17E precursor | Q9H293-2 | N-linked | yes |
| Splice Isoform 2 Of Phospholipid transfer protein precursor | P55058-2 | N-linked | yes |
| Splice Isoform 2 Of Poliovirus receptor related protein 1 precursor | Q15223-2 | N-linked | yes |
| Splice Isoform 2 Of Voltage-dependent N-type calcium channel alpha-1B subunit | Q00975-2 | N-linked | ? |
| Splice Isoform 3 Of Neuronal cell adhesion molecule precursor | Q92823-3 | N-linked | yes |
| Splice Isoform 3 Of Neurotrimin precursor | Q9P121-3 | N-linked | yes |
| Splice Isoform 4 Of Neuronal cell adhesion molecule precursor | Q92823-4 | N-linked | yes |
| Splice Isoform 7 Of Myelin-oligodendrocyte glycoprotein precursor | Q16653-7 | N-linked | ? |
| Taste receptor type 2 member 16 | Q9NYV7 | N-linked | yes |
| Taste receptor type 2 member 7 | Q9NYW3 | N-linked | ? |
| Transthyretin precursor | P02766 | N-linked | yes |
| Tumor necrosis factor receptor superfamily member 8 precursor | P28908 | N-linked | ? |
| Vitamin D-binding protein precursor | P02774 | N-linked | yes |
| Vitamin K-dependent protein S precursor | P07225 | N-linked | yes |
| Vitronectin precursor | P04004 | N-linked | yes |
| Ceruloplasmin precursor | P00450 | N-linked | yes |
| NeuroNal peNtraxiN receptor isoform 1 | O95502 | N-linked | yes |
| Splice Isoform 3 Of Seizure 6-like protein precursor | Q9BYH1 | N-linked | ? |
| Complement C1q subcomponent, C chain precursor | P02747 | O-linked | yes |
| Secretogranin I precursor | P05060 | O-linked | yes |
| Ubiquitin carboxyl-terminal hydrolase isozyme L1 | P09936 | O-linked | ? |
| Nogo receptor-like 3 | Q86UN3 | GPI anchored | yes |
| AMBP protein precursor | P02760 | N,O-linked | yes |
| Amyloid-like protein 1 precursor | P51693-1 | N,O-linked | yes |
| Apolipoprotein E precursor | P02649 | N,O-linked | yes |
| Beta-2-glycoprotein I precursor | P02749 | N,O-linked | yes |
| Coagulation factor XII precursor | P00748 | N,O-linked | yes |
| Collagen alpha 1(I) chain precursor | P02452 | N,O-linked | yes |
| Hemopexin precursor | P02790 | N,O-linked | yes |
| Inter-alpha-trypsin inhibitor heavy chain H2 precursor | P19823 | N,O-linked | yes |
| Leucine-rich alpha-2-glycoprotein precursor | P02750 | N,O-linked | yes |
| Lysosomal-associated membrane protein 2C | P13473 | N,O-linked | yes |
| Mimecan precursor | P20774 | N,O-linked | yes |
| Phosphatidylcholine-sterol acyltransferase precursor | P04180 | N,O-linked | yes |
| Pigment epithelium-derived factor precursor | P36955 | N,O-linked | yes |
| Plasma protease C1 inhibitor precursor | P05155 | N,O-linked | yes |
| Plasminogen precursor | P00747 | N,O-linked | yes |
| Serotransferrin precursor | P02787 | N,O-linked | yes |
| SPARC-like protein 1 precursor | Q14515 | N,O-linked | yes |
| Splice Isoform 1 Of Brevican core protein precursor | Q96GW7-1 | N,O-linked | yes |
| Splice Isoform 1 Of Ecto-ADP-ribosyltransferase 3 precursor | Q13508-1 | N,O-linked | yes |
| Splice Isoform 1 Of Inter-alpha-trypsin inhibitor heavy chain H4 precursor | Q14624-1 | N,O-linked | yes |
| Splice Isoform 2 Of Kininogen precursor | P01042-2 | N,O-linked | yes |
| Splice Isoform 3 Of Amyloid beta A4 protein precursor | P05067-3 | N,O-linked | yes |
| Splice Isoform 3 Of Fibronectin precursor | P02751-3 | N,O-linked | yes |
| Splice Isoform 4 Of Fibronectin precursor | P02751-4 | N,O-linked | yes |
| Splice Isoform 5 Of Amyloid beta A4 protein precursor | P05067-5 | N,O-linked | yes |
| Tyrosine phosphatase zeta polypeptide 2 HTPZP2 | P23471 | N,O-linked | yes |
| Monocyte differentiation antigen CD14 precursor | P08571 | N,O-linked, GPI anchored | yes |
| 39 kDa protein | Q7Z3B1 | N-linked,GPI-anchored | yes |
| CD59 glycoprotein precursor | [P13987](http://srs.ebi.ac.uk/srsbin/cgi-bin/wgetz?-id+4xQIW1hZBXi+%5Bswissprot-AccNumber:P13987%5D+-e) | N-linked,GPI-anchored | yes |
| Contactin 2 precursor | Q02246 | N-linked,GPI-anchored | yes |
| Ephrin A1 isoform b precursor | P20827-2 | N-linked,GPI-anchored | yes |
| Limbic system-associated membrane protein precursor | Q13449 | N-linked,GPI-anchored | yes |
| Semaphorin 7A precursor | O75326 | N-linked,GPI-anchored | yes |
| Inter-alpha-trypsin inhibitor heavy chain H1 precursor | P19827 | S,N,O-linked | yes |
| Complement component C6 precursor | P13671 | C,N,O-linked | yes |
| Complement component C7 precursor | P10643 | C,N,O-linked | yes |
